# Closing the HNDL Window in Consumer eSIM Provisioning: Hybrid Post-Quantum Migration, Formal Verification, and Deployment Constraints on eUICC Silicon

**DOI:** 10.3390/s26154683

**Published:** 2026-07-23

**Authors:** Jhury Kevin Lastre, Yongho Ko, Hoseok Kwon, Ilsun You

**Affiliations:** 1 Department of Information Security, Cryptology, and Mathematics, Kookmin University, Seoul 02707, Republic of Korea; lavelliane@kookmin.ac.kr (J.K.L.); koyh0911@kookmin.ac.kr (Y.K.); hoseok1997@kookmin.ac.kr (H.K.); 2 KMU Global Research Center for ICT Convergence Security, Kookmin University, Seoul 02707, Republic of Korea

**Keywords:** post-quantum cryptography, ML-KEM, ML-DSA, eSIM, SGP.22, remote SIM provisioning, formal verification, ProVerif, harvest-now–decrypt-later, hybrid key exchange

## Abstract

Embedded Subscriber Identity Modules (eSIMs) enable consumer devices to install mobile subscriptions remotely under the GSMA SGP.22 standard for Remote SIM Provisioning (RSP). Because RSP sessions rely on classical elliptic-curve cryptography and eSIM profiles can remain active for 5 to 20 years, recorded provisioning traffic faces a concrete Harvest-Now–Decrypt-Later (HNDL) threat. Upgrading the network transport to post-quantum Transport Layer Security (TLS) is often assumed to be sufficient. However, SGP.22 exchanges the keys that protect the profile across the local host-to-chip interface, beneath the transport layer. This paper presents a systematic post-quantum cryptography (PQC) migration framework for consumer RSP. We model four configurations of the SGP.22 on-card key-agreement step and determine, under a quantum key-recovery adversary, which configurations resist HNDL and what resources they require. We combine symbolic verification in ProVerif with a device-grounded evaluation that pairs provisioning and memory observations from a sysmocom C2T research embedded Universal Integrated Circuit Card (eUICC) with strict-instruction-set PQC measurements on an STM32 Nucleo-F446RE development board with an ARM Cortex-M4F core. Among the configurations studied, hybrid classical and post-quantum key exchange is the minimum configuration that resists HNDL, whereas a fully post-quantum configuration also protects authentication against signature forgery. Under the tested platform and resource assumptions, volatile Random Access Memory (RAM), rather than computation, is the binding deployment constraint. We therefore propose a capability-negotiation mechanism that would match a migration configuration to the memory advertised by each card.

## 1. Introduction

Modern smartphones, tablets, wearables, connected vehicles, and a growing population of Internet of Things (IoT) devices rely on embedded Subscriber Identity Modules (SIMs), commonly called eSIMs. An eSIM is a subscriber identity chip built into the device rather than a removable plastic card. Instead of inserting a card, a network operator can install a subscription, called a *profile*, over the internet through Remote SIM Provisioning (RSP). For consumer devices, RSP is governed by the GSM Association (GSMA) specification SGP.22 [1], which provides the standard process for securely installing a subscription profile on a device already in a user’s hands.

Like most secure communication today, SGP.22 protects each provisioning session with classical public-key cryptography, principally Elliptic-Curve Diffie–Hellman (ECDH) key exchange and elliptic-curve digital signatures. A sufficiently powerful quantum computer, that is, a cryptographically relevant quantum computer (CRQC), will eventually break this cryptography using Shor’s algorithm [2]. The immediate danger lies in today’s traffic, not tomorrow’s. In a Harvest-Now–Decrypt-Later (HNDL) attack [3], an adversary records protected provisioning traffic now and decrypts it once a CRQC becomes available [2,4]. eSIM profiles can remain active for the 5-to-20-year lifetime of a device and protect long-lived subscriber credentials. Consequently, the HNDL exposure window is wide, and, under Mosca’s theorem [5], the migration deadline for the longest-lived deployments has already arrived.

The difficulty is that the vulnerable keys do not remain solely within the protected network connection to the operator. SGP.22 exchanges the ephemeral keys that derive the profile-encryption key across the local link between the device’s host software and its secure-element chip, beneath the transport layer. Upgrading only the network transport to post-quantum Transport Layer Security (TLS), therefore, leaves these keys exposed on the local interface. Closing the HNDL window requires a cryptographic change to the on-card key agreement itself. This setting is highly constrained: secure elements have small processor cores and only kilobytes of working memory, and SGP.22 provides no way to match a migration configuration to a card’s available resources.

To the best of our knowledge, prior work on consumer RSP has not combined an SGP.22-specific quantum-adversary analysis with device-grounded resource evidence for on-card migration. Existing consumer-RSP verification assumes classical cryptography; post-quantum eSIM work studies secure-channel protocols rather than the SGP.22 profile-protection key agreement; and most implementation evidence comes from microcontroller benchmarks rather than eUICC provisioning measurements (Section 2). Industry guidance recommends hybrid post-quantum migration at a general level but does not determine which SGP.22 configuration closes the local-interface HNDL exposure or what resources that configuration requires. The resulting questions are which configuration closes the HNDL window, what constraints appear on the tested C2T and STM32 platforms, and what additional evidence is required before broader deployment claims can be made.

This paper addresses these questions through a systematic PQC migration framework for consumer RSP. We map the SGP.22 design space as four configurations of the on-card key agreement and determine which configurations close the HNDL window and what resources they require. The framework is supported by two complementary forms of evidence. First, we use a ProVerif model of SGP.22 authentication and download procedures under a quantum-capable adversary. Second, we combine provisioning and RAM observations from a research eUICC with PQC cycle and memory measurements from an embedded development board. Under these platform-specific assumptions, the analyses identify volatile RAM, rather than computation, as the binding constraint. They also motivate a proposed capability-negotiation mechanism for matching migration configurations to advertised card resources.

**Contributions.** Specifically, this paper makes the following contributions.

**C1.**  *A post-quantum migration framework for consumer RSP.* We organize the SGP.22 migration as four configurations of the on-card key agreement, identify the hybrid configuration as the minimum configuration that closes the HNDL window, and define the fully post-quantum configuration as the target state. We package these results as a phased migration path with capability negotiation for deployment across cards with different memory resources (Section 4 and Section 6).
**C2.**  *Formal verification as supporting evidence.* A ProVerif model under a quantum key-recovery adversary shows why post-quantum TLS alone does not close the HNDL window and why the hybrid on-card key agreement does (Section 4).
**C3.**  *Device-grounded resource analysis as supporting evidence.* Research-eUICC provisioning and RAM observations, combined with STM32 PQC cycle and memory measurements, identify volatile RAM as the binding constraint under the tested assumptions (Section 5). The resulting capability negotiation is a protocol-design proposal, not a standardized or deployed mechanism.

We do not propose a new cryptographic primitive; hybrid key agreement and its security are well-established (Section 2). Our contribution lies in connecting an SGP.22-specific HNDL verdict to research-card provisioning baselines, strict-instruction-set embedded measurements, and explicit resource projections. This integrated perspective uncovers a volatile-RAM constraint under the tested assumptions that transport-level and primitive-level analyses do not expose by themselves. We label each claim as Proven, Measured, Derived, Projected, or Recommended to make the evidence chain explicit (Section 5.5).

The remainder of this paper is organized as follows. Section 2 surveys prior work along three axes: formal verification of cellular and eSIM protocols, PQC on constrained embedded hardware, and PQC migration standards. Section 3 reviews the SGP.22 architecture, pinpoints the HNDL exposure, defines the post-quantum primitives and the threat model, and specifies the four migration configurations. Section 4 presents the formal verification. Section 5 reports the evaluation using a research eUICC and an embedded development board. Section 6 derives the RAM-aware, phased migration path. Section 7 states the limitations and future work, and Section 8 concludes the article.

## 2. Related Work

Three axes of prior work are directly relevant: (i) formal verification of cellular and mobile protocols, (ii) PQC performance on constrained embedded hardware, and (iii) PQC migration standards and policy.

### 2.1. Formal Verification of Cellular and eSIM Protocols

Symbolic verification under Dolev–Yao adversaries [6] via ProVerif [7,8] in the applied pi-calculus [9] and Tamarin is the established tool family for cellular and mobile protocols. Recent examples include Cremers et al. [10] on TLS 1.3 (informing RFC 8446 [11]) and Basin et al. [12] together with Hussain et al. [13] on 5G-AKA (Authentication and Key Agreement). Damir et al. [14] apply ProVerif to a PQ-hybrid 5G-AKA protocol (the closest cellular methodological precedent, scoped to radio-side authentication rather than RSP), and Bhargavan et al.’s ProVerif and CryptoVerif analysis of Signal PQXDH [15] models the classical-component break via compromise of long-term identity keys, showing that Key Encapsulation Mechanism (KEM)-backed secrecy survives. Our SGP.22 model adapts that classical-side-break framing as an *ephemeral-compromise analog*. The SGP.22 attacker harvests ephemeral Application Protocol Data Unit (APDU) public keys rather than receiving long-term identity-key reveals, which we encode through an unconditional break_dh equation. The modeling details of the equation and its per-trace seen_pk annotations are in Section 4. Within eSIM, Ahmed et al. [16] give the closest prior consumer-RSP analysis but under classical assumptions. Ko et al. [17] cover SGP.02 Machine-to-Machine (M2M). Lastre et al. [18] verify SGP.22 mutual authentication via BAN (Burrows–Abadi–Needham) logic. Bettale et al. [19] formally verify an eSIM *secure-channel* variant in ProVerif under a classical (non-quantum) adversary, which complements rather than overlaps with our break_dh-extended RSP model (Section 4.1) with an oracle-fire diagnostic. To our knowledge, no prior work targets a quantum attacker at the SGP.22 RSP key exchange itself, which is the gap that this paper closes.

### 2.2. PQC Performance on Constrained Embedded Hardware

pqm4 [20] is the canonical Cortex-M4 benchmarking framework for National Institute of Standards and Technology (NIST) PQC candidates, and PQClean [21] complements it with portable-C reference implementations for the ARMv7-M subset shared with the tested SC300 target. Greconici et al. [22] give closely related implementation evidence for a DSP-less Cortex-M3, confirming that lattice schemes run on this instruction-set class at a measurable penalty. Published PQC-performance evidence remains concentrated on Cortex-M development boards rather than eUICC deployments. This study does not close that gap with direct on-card PQC execution. Instead, it combines C2T provisioning and RAM observations with strict ARMv7-M measurements on the STM32 to narrow the resource question for the tested target. The fact that lattice KEMs can run faster than ECDH P-256 on ARM hardware is established by independent studies (Table 1). Saarinen [23] measured 5.6× lower energy on Cortex-M4 for Kyber-768 compared with ECDH P-256, wolfSSL benchmarks [24] show that Kyber decapsulation is roughly 3× faster than ECDH secp256r1 agreement at 168 MHz, and Kannwischer et al. [20] report a large pqm4 cycle advantage. Our contribution relative to this record is a per-innermost-multiply audit and a strict ARMv7-M re-measurement that tests instruction-set portability relevant to the SC300 target. Chip-faithful on-card PQC timing remains open.

### 2.3. PQC Migration Standards and Policy

NIST standardized ML-KEM as FIPS 203 [25] and ML-DSA as FIPS 204 [26]. SP 800-131A [27], together with the recent NIST IR 8547 [28], earmarks 2030 as the ECDH and Elliptic Curve Digital Signature Algorithm (ECDSA) deprecation milestone, with disallowance by 2035. SP 800-227 [29] covers KEM key establishment, and the two-input concatenation Key-Derivation Function (KDF) that combine_kdf instantiates follows the companion SP 800-56C Rev.2 [30]. The 3rd Generation Partnership Project (3GPP) TR 33.841 [31] extends the scope to 5G with the 256-bit and quantum-safe algorithm study, sharing the long-lived subscriber-credential surface that SGP.22 RSP also defends. The hybrid-KEM design principle for TLS is from Bos et al. [32], and the combiner-level indistinguishability result is from Bindel et al. [33], with the construction subsequently underpinning Internet Engineering Task Force (IETF) drafts such as X25519Kyber768 [34]. GSMA PQ.03 [35] recommends hybrid post-quantum cryptography as the transitional migration approach for telecom use cases (a guideline-level rather than normative document), discussing security levels, performance impact, and deployability without SGP.22-specific HNDL proof or a research-eUICC resource study. The companion GSMA PQ.04 [36] extends the analysis to the IoT ecosystem (SGP.31, SGP.32), where 10–20-year device lifetimes amplify the HNDL harvest window further. Adjacent telco results (5G-AKA [37], TLS 1.3 roaming [38]) and the constrained-IoT RAM-barrier observation of Kumar et al. [39] are the operational anchors we extend to SGP.22.

Table 2 separates the established components from the SGP.22-specific integration studied here. Ahmed et al. [16] already provide a detailed ProVerif model of consumer RSP, but under classical and partial-compromise adversaries. Bettale et al. [19] provide post-quantum secure-channel variants for eSIM application management, together with formal and chip-level evidence, but do not analyze the SGP.22 profile-protection key agreement under local-interface HNDL. The remaining sources establish the hybrid construction, the embedded implementation baseline, or the migration guidance used in this paper.

The residual contribution is therefore the SGP.22-specific evidence chain: it connects local-APDU exposure under HNDL, four on-card migration configurations, symbolic wire-format verification, platform-bounded resource evidence, and a backward-compatible capability-negotiation proposal. The formal verification and performance components support this system-level result; they are not presented as new primitive-level theorems or as vendor-neutral hardware characterization.

## 3. SGP.22 HNDL Exposure and the PQC Migration Design Space

### 3.1. SGP.22 Architecture

Three actors participate in every consumer-RSP session. The eUICC is a secure element on the consumer device storing eSIM profiles in non-volatile memory (NVM). The SM-DP+ server, operated by a mobile operator or profile vendor, prepares and delivers encrypted profile packages. The Local Profile Assistant (LPA) is host-side software that mediates between the two [1,41]. Two channels carry protocol messages. The *APDU channel* (ISO 7816/PC/SC [42]) between the LPA and the eUICC is a local, unprotected bus. The *ES9+ channel* (Hypertext Transfer Protocol Secure, HTTPS) between the LPA and the SM-DP+ is protected by TLS 1.3 [11] in config. (a) and by post-quantum TLS (PQ-TLS) in configs. (b)–(d). A four-level certificate hierarchy anchors trust at the GSMA CI, where the SM-DP+ holds a CI-signed DPauth certificate and the eUICC holds a CI-signed eUICC Manufacturer (EUM) certificate. The session-key agreement uses ephemeral ECDH key pairs, newly generated each session. The architectural separation of the two channels has a security consequence, which is analyzed throughout this paper.

### 3.2. Authentication and Download Phases

**Figure 1:**
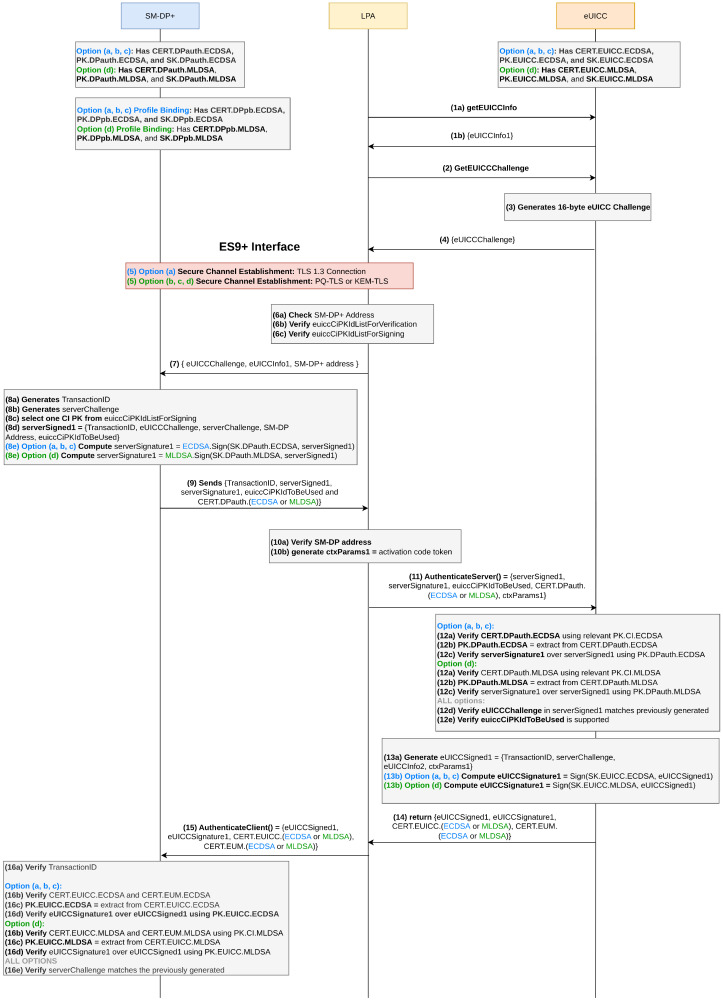
SGP.22 authentication phase (steps 1–16). Blue text marks steps specific to configurations (a), (b), and (c); green text marks steps specific to configuration (d); the red box marks the ES9+ secure-channel establishment (step 5).

Figure 1 shows the SGP.22 mutual authentication protocol (steps 1–16, Section 3.1).

The SM-DP+ generates an ephemeral ECDH key pair and delivers otPK.DP.ECKA inside the signed smdpSigned2 payload of the BF21 PrepareDownload APDU command at step 19. The eUICC generates its own ephemeral ECDH pair on receipt and returns otPK.EUICC.ECKA inside the signed euiccSigned2 structure, transmitted in the BF22 PrepareDownload response at step 22.

**Figure 2:**
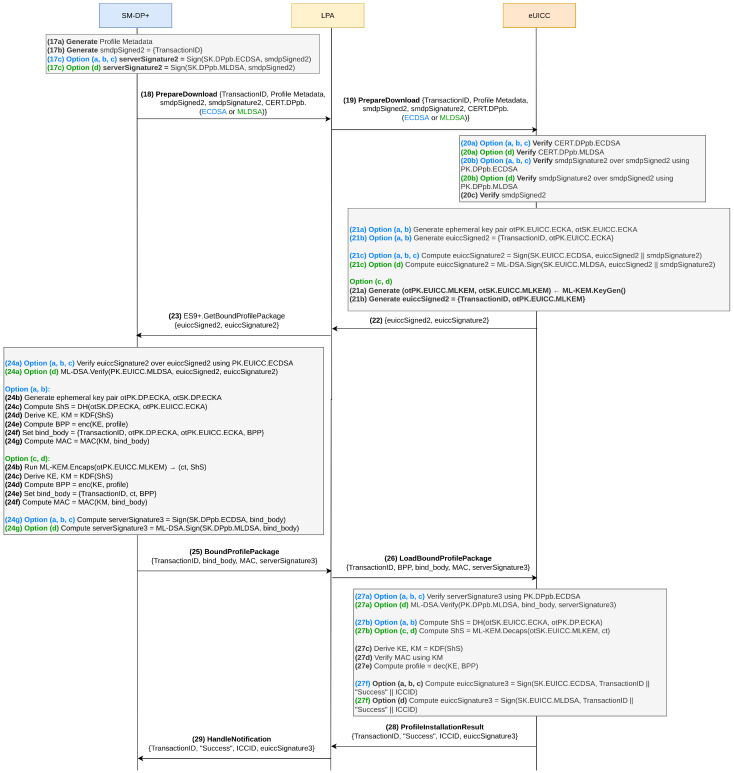
SGP.22 profile download phase (steps 17–29). The BF21 PrepareDownload command is the step-27b key-derivation point where the four configurations diverge. Blue text marks steps specific to configurations (a), (b), and (c); green text marks steps specific to configuration (d).

Figure 2 shows the profile download protocol (steps 17–29, Section 3.1).

The BF21 PrepareDownload command is the key-derivation step at which the eUICC uses the two ephemeral ECDH public keys to derive the BPP encryption key $K_{E}$ and integrity key $K_{M}$ via a KDF. For configs. (b)–(d) the same step also involves ML-KEM-768 material (Section 3.5). The BF36 LoadBoundProfilePackage command delivers the Advanced Encryption Standard 128-bit Cipher Block Chaining (AES-128-CBC) BPP-encrypted profile in T = 0 ISO 7816 blocks of 255 bytes each. The eUICC decrypts these blocks in constrained on-card hardware, and this step dominates the session (13.839 s or 53%, as reported in Section 5.2).

The APDU channel carries both ephemeral ECDH public keys in plaintext regardless of the ES9+ transport, an architectural property of SGP.22 that is the mechanistic basis for the config. (a)/config. (b) HNDL FAIL formalized in Section 4.

### 3.3. Post-Quantum Primitives

Two lattice-based primitives are used throughout the migration configurations. Neither problem is known to admit a polynomial-time quantum algorithm. Shor’s algorithm applies to the hidden subgroup problem over abelian groups (covering RSA and elliptic-curve discrete logarithm) but not to the lattice problems underlying ML-KEM-768 and the Module-Lattice-Based Digital Signature Algorithm (ML-DSA-44). The migration studied here is therefore entirely post-quantum (software lattice cryptography), as designated in the GSMA PQ.03/PQ.04 and NIST migration roadmaps, rather than quantum cryptography, such as quantum key distribution (QKD). QKD and the in-band post-quantum alternatives are discussed separately in Section 6.7.

#### 3.3.1. ML-KEM-768

ML-KEM-768 [25] is a Key Encapsulation Mechanism based on the Module Learning With Errors (MLWE) problem over the polynomial ring $R_{q}=Z_{q}\left[x\right]/\left(x^{n}+1\right)$, with n=256, q=3329, k=3, and η=2, targeting NIST security level 3 and referenced as the recommended post-quantum KEM in the GSMA telecom migration guidelines (PQ.03 [35]). We adopt the standard FIPS 203 procedures verbatim. KeyGen produces (ek,dk), with |ek|=1184 B and |dk|=2400 B. Encaps (called by the SM-DP+ at SGP.22 step 21a) yields a shared key *K* (32 B) and a ciphertext *c* (1088 B). Decaps (called by the eUICC at step 27b) returns *K* with the Fujisaki–Okamoto implicit-rejection check, which is the reason Decaps requires slightly more cycles than Encaps. The Number Theoretic Transform (NTT) over q=3329 satisfies q≡1(mod2n), so polynomial multiplication decomposes into in-place butterflies of modular additions, subtractions, and twiddle multiplications. Every intermediate coefficient fits in 14 bits and requires no 64-bit carry chain. This structural property is the mechanistic origin of Finding A (Section 5.3).

#### 3.3.2. ML-DSA-44

ML-DSA-44 [26] is a Module Short Integer Solution (MSIS)/MLWE-based signature scheme. ML-DSA-44 (NIST level 2) is paired with ML-KEM-768 (level 3) deliberately. Under HNDL the load-bearing primitive is the KEM (guarding year-scale BPP confidentiality), while the signature gates only session-bound authentication, which has no HNDL exposure, so the Cat-2 strength of ML-DSA-44 (categorized in FIPS 204 §1.2 and Table 1) is adequate for our threat model. The signature size (2420 B vs. 3309 B for ML-DSA-65) dominates the BF38 bind_body budget (the signed data structure over which the SM-DP+ binds the profile package to the session), driving Finding B. The integration is algorithm-agnostic at the symbolic layer and could substitute for ML-DSA-65 at the BF38 slot without affecting the verdict. Falcon-512 would halve the signature size further, but its floating-point Gaussian sampler is hostile to constant-time implementation on SC300, and integer-only Falcon variants under active standardization remain pre-deployment. We adopt FIPS 204 verbatim. KeyGen produces (vk,sk), with |vk|=1312 B and |sk|=2560 B; Sign is Fiat–Shamir with aborts (expected mean ≈4.25 rejection iterations per FIPS 204 §C.3); and the signature $\sigma =(\tilde{c},z)$ is 2420 B (37.8× an ECDSA P-256 signature).

#### 3.3.3. Key Size Comparison

Table 3 summarizes the key material sizes that drive Finding B. The 17- to 38-fold increase inflates every protocol message that carries key-exchange data, and whether SGP.22 sessions can absorb this inflation is the empirical question addressed in Section 5.4.

### 3.4. The Local-APDU HNDL Exposure and Quantum Attacker Model

The threat model is a Dolev–Yao attacker [6] augmented with a single quantum oracle:

(1) $$\mathrm{break}\text{\_}\mathrm{dh}\left(pk_{A},\;pk_{B}\right)\;\to \;\mathrm{shS}=\mathrm{ECDH}\left(sk_{A},\;pk_{B}\right),$$

modeling Shor’s algorithm applied retroactively to recorded APDU traffic. The equation is unconditional: it admits inversion on any two ecpoint inputs the attacker can synthesize, which makes the symbolic adversary strictly stronger than a realistic harvester and therefore renders the positive verdicts (Q5/Q6 TRUE) conservative. A diagnostic query QD = query attacker(shS_1) reports whether the ECDH-branch shared secret of config. (c) is recoverable. For config. (c), we report QD = FALSE (${\mathrm{shS}}_{1}$ is indeed recovered, as expected under the equation) together with Q5 = TRUE (the session key remains secret), showing that combine_kdf hides *K* under partial quantum compromise so long as the MLWE-protected ${\mathrm{shS}}_{2}$ branch holds.

**Computational assumptions.** The analysis rests on six assumptions, each gating a specific part of the verdict (Table 4).
**Attacker boundary and trusted components.** The attacker controls ES9+ fully, passively reads the APDU channel, and possesses the break_dh oracle but not break_kem or break_sign. Side channels, fault injection, and physical key extraction are out of scope. The GSMA CI and its Public-Key Infrastructure (PKI) are uncompromised (**T1**), ephemeral keys are Cryptographically Secure Pseudorandom Number Generator (CSPRNG)-generated (**T2**), and the model captures protocol logic, not implementation bugs (**T3**). The passive PC/SC observer collapses several deployment-realistic positions to the same channel-observation power: host-side LPA malware with permissive pcscd privileges, hardware reader-tap at retail provisioning, and long-lived embedded eUICCs (automotive, industrial-gateway, connected-medical) where 10–20-year profile-binding lifetimes coincide with physical accessibility during service or salvage. The adversary needs no live presence when Shor’s algorithm becomes available, only a recorded APDU trace. Documented real-world eUICC compromises that extract on-card identity material and download profiles in cleartext corroborate that host-side and reader-side access to the local interface is a realistic adversarial vantage rather than a hypothetical one [43].
**Physical-layer threats (out of symbolic scope).** Side-channel analysis, fault injection, and physical key extraction are outside the symbolic threat model and are not evaluated experimentally in this study. Their implications for hardened implementations are treated as literature-derived projections and future work in Section 7.
**Harvest frequency and the symmetric floor.** Because the break_dh oracle is unconditional, a harvested session is recovered deterministically once observed rather than with some per-attempt probability, so the rate of provisioning scales the *number* of independent HNDL harvest opportunities linearly (each session re-emits both ephemeral ECDH public keys on the cleartext PC/SC bus) rather than a per-attempt success probability. Symmetric-key strength enters separately: under A5, the AES-128 BPP floor falls to approximately $2^{64}$ under Grover, one level below ML-KEM-768’s NIST Category-3 (AES-192) target, so the public-key migration inherits this floor rather than raising it, and a 256-bit BPP cipher upgrade is an orthogonal specification change (Section 8).

### 3.5. The Four Migration Configurations

Table 5 defines the four configurations studied throughout the paper, from the deployed baseline config. (a) through the PQ-TLS-only config. (b) and the hybrid step-27b config. (c) [32,34,35,40] to the fully post-quantum config. (d). The four step-27b key derivations, each expanding the shared secret through the Hash-based Message Authentication Code (HMAC), specifically the HMAC-based Key-Derivation Function (HKDF), are compactly

$$\begin{array}{cc} (a),(b):\; & K_{E},K_{M}=\mathrm{HKDF}\left(\text{ECDH.Agree}(sk,otPK)\right)\\ (c):\; & K_{E},K_{M}=\mathrm{HKDF}\left(\mathrm{combine}\text{\_}\mathrm{kdf}({\mathrm{shS}}_{1},{\mathrm{shS}}_{2})\right)\\ \; & \;\;{\mathrm{shS}}_{1}=\text{ECDH.Agree}\left(sk,otPK\right),\\ \; & \;\;{\mathrm{shS}}_{2}=\text{ML-KEM-768.Decaps}\left(dk,ct\right)\\ (d):\; & K_{E},K_{M}=\mathrm{HKDF}\left(\text{ML-KEM-768.Decaps}(dk,ct)\right),\\ \; & \;\;\mathrm{auth}\;\mathrm{via}\;\text{ML-DSA-44} \end{array}$$

In config. (c), the break_dh oracle may recover ${\mathrm{shS}}_{1}$ but not ${\mathrm{shS}}_{2}$ (MLWE-hard), and under A6 the combine_kdf output is then indistinguishable from uniform. In config. (d), no ECDH operation exists, so break_dh is vacuously inapplicable.

**Combiner choice.** Config. (c) implements the standard-model hybrid construction of Bos et al. [32] and Giacon, Heuer, and Poettering [40] (also the IETF X25519Kyber768 design [34] and the transitional telecom approach of GSMA PQ.03 [35]): a classical and a post-quantum key exchange run in parallel, and their shared secrets are combined through a KDF that remains indistinguishable from uniform when either input is hidden. Specifically, combine_kdf $\left(s_{1},s_{2}\right)$ is HKDF-Extract (HMAC-based Key-Derivation Function) over the concatenation $s_{1}∥s_{2}$ with a fixed salt, in alignment with the concatenation KDF of SP 800-56C Rev.2 [30], and is therefore a pseudorandom function (PRF) under A6, which is exactly the premise of the either-input-hidden combiner security of Giacon, Heuer, and Poettering [40] and Bindel et al. [33]. Because the model abstracts the combiner as an opaque PRF under A6, the HNDL verdict is agnostic to the specific KDF and scales across combine_kdf, X-Wing, and the other standard-model KEM combiners, trading off only computational-level binding guarantees, not the symbolic HNDL property (Section 4.5). The X-Wing combiner [44] additionally provides MAL-BIND-K-PK and MAL-BIND-K-CT robustness [45] by binding both ECDH public keys and the ML-KEM-768 ciphertext into the KDF. SGP.22 does not match this KDF-layer property primitively, but the outer SM-DP+ ML-DSA-44 or ECDSA P-256 signature over bind_body covers the same fields at the protocol layer before session-key acceptance, which we treat as a wire-level compensation; we therefore retain combine_kdf, consistent with the GSMA guidelines that recommend hybrid migration without mandating a combiner (PQ.03 [35], PQ.04 [36]). This compensation is horizon-dependent: because the configuration-(c) bind_body signature is ECDSA P-256, it degrades under the long-term break_ecdsa adversary of Section 6.6, where X-Wing’s KDF-internal binding would survive, so a deployment that must resist cross-component binding against a signature-forging quantum adversary should prefer X-Wing or move to configuration (d).

## 4. Formal HNDL Resistance

### 4.1. Symbolic Model and Queries

We model all four configurations in ProVerif 2.05 [7,8] using the applied pi-calculus [9]. The public channel ch represents the APDU channel observable by the network attacker. The ES9+ channel is modeled as private in configs. (b)–(d) and as public in config. (a). Long-term private keys are ProVerif private names inaccessible to the attacker, and ephemeral keys are newly generated each session using new. The quantum oracle (Equation (1)) is implemented as an unconditional ProVerif equation available to the attacker on any two ecpoint inputs it can synthesize (an over-approximation). The diagnostic QD is a separate secrecy query, query attacker(shS_1), on the intermediate ECDH-branch shared secret of config. (c). Table 6 summarizes the eight security queries plus the diagnostic.

### 4.2. Security Requirements and Their ProVerif Encoding

Authentication-style requirements (Q1–Q4, Q7) are evaluated through injective correspondence queries (inj-event) so that each accepted message uniquely corresponds to an authentic transmission, while confidentiality-style requirements (Q5, Q6) use secrecy queries over private-channel data. Q8 is deliberately *classical* post-compromise secrecy (classical PCS) rather than HNDL resistance, combining the secrecy queries with a post-session long-term signing-key disclosure phase (phase 1) and no break_dh oracle. HNDL resistance is evaluated separately under the break_dh-extended attacker, with QD reporting whether the ECDH-branch shared secret ${\mathrm{shS}}_{1}$ is attacker-recoverable on config. (c) under the equation (it is, as expected, and the Q5/Q6 verdict on config. (c) then says combine_kdf still hides the session key). The concrete queries are summarized in Table 6.

### 4.3. Applied Pi-Calculus Model

The four configurations share one model and diverge only at the key-exchange and authentication step. All use a public APDU channel ch_apdu and a private ES9+ channel ch_es9, emit correspondence events S_·/E_· on send and receive for the injective authentication queries Q1–Q4, and are analyzed under the break_dh oracle of Equation (1) against the queries of Table 6. Algorithms 1–4 give the compact eUICC process for each configuration, with a specific color marking its distinctive property (the SM-DP+ side is structurally symmetric); the main process composes the roles under unbounded replication and stages a post-session long-term-secret disclosure phase (phase 1) for forward secrecy.
**Algorithm 1** config. (a), classical baseline: ECDSA P-256 authentication, ECDH P-256 key exchange, TLS 1.3 transport. Red marks the values that a break_dh harvester recovers. eUICC side; SM-DP+ symmetricnew x; X = gˆx; event seen_pk(X); out(ch_apdu, X)   (* ECDH public sent in cleartext *)in(ch_apdu, (Y, cert)); verify(cert);   shS = ECDH.Agree(x, Y)   (* break_dh recovers shS *)K_E, K_M = HKDF(shS);      ES9+: TLS 1.3.**Verdict:** HNDL-vulnerable; Q5, Q6 FAIL.

**Algorithm 2** config. (b), PQ-TLS transport only: the eUICC key exchange is identical to that in config. (a). Blue marks the sole change; red, the still-harvestable share
(* eUICC key exchange exactly as in config. (a): *) shS = ECDH.Agree(x, Y); K_E, K_M = HKDF(shS).ES9+: PQ-TLS   (* the only difference from config. (a) *)**Verdict:** Still HNDL-vulnerable: the ECDH share sits below the transport, so PQ-TLS does not protect it; Q5, Q6 FAIL.


The queries instantiate Q1–Q8 and QD as injective correspondences (Q1–Q4, Q7) and secrecy queries (Q5, Q6, Q8, QD). The break_dh oracle is unconditional: it admits inversion on any two public-key inputs the attacker can synthesize, which makes the positive verdicts conservative. Each honest out(ch_apdu,…) site is instrumented with a seen_pk event, a trace-inspection aid in the style of [10,14] that does not constrain the oracle but lets the reader confirm that ProVerif-reported attack traces use public keys that actually traversed ch_apdu. QD = FALSE on config. (c) reports that ${\mathrm{shS}}_{1}$ is indeed recovered (the ECDH branch is broken), and the corresponding Q5 = TRUE then says that combine_kdf hides *K* because ${\mathrm{shS}}_{2}$ remains MLWE-protected under A2. Q8 is verified by a phase-1 disclosure of long-term signing keys, separating classical post-compromise secrecy from the HNDL oracle analysis.
**Algorithm 3** config. (c), hybrid ECDH P-256  ‖ ML-KEM-768 with combine_kdf. Green marks the post-quantum branch that closes HNDL; red, the ECDH branch the oracle still breaksnew x; X = gˆx; new dk; ek = ML-KEM-768.ek(dk); out(ch_es9, ek);event seen_pk(X); out(ch_apdu, X); in(ch_apdu, (Y, cert)); in(ch_es9, ct);shS1 = ECDH.Agree(x, Y)   (* break_dh recovers shS1 *)shS2 = ML-KEM-768.Decaps(dk, ct)   (* MLWE-hard: not recoverable *)K = combine_kdf(shS1, shS2); K_E, K_M = HKDF(K).**Verdict:** HNDL-resistant; Q5, Q6 hold; QD: the oracle fires but is blocked.

**Algorithm 4** config. (d), full post-quantum: ML-DSA-44 authentication, ML-KEM-768 key exchange, no ECDH. Green marks the post-quantum primitives that make break_dh inapplicable
new dk; ek = ML-KEM-768.ek(dk); out(ch_es9, ek);in(ch_apdu, (cert_DP, sig)); if ML-DSA-44.Verify(cert_DP, sig);in(ch_es9, ct); shS = ML-KEM-768.Decaps(dk, ct)   (* no ECDH at all *)K_E, K_M = HKDF(shS).**Verdict:** HNDL-resistant; break_dh vacuously inapplicable; Q1–Q7 hold.


### 4.4. Classical-Baseline Verification Results

Under the classical Dolev–Yao attacker (no break_dh oracle), all nine queries return TRUE for all four configurations, establishing that subsequent Q5/Q6 failures under the quantum oracle are caused by the oracle itself rather than a pre-existing weakness.

### 4.5. Quantum-Adversary Results: The Hybrid Is the Minimum HNDL-Resistant Configuration

Table 7 shows the oracle-scenario results.

**Figure 3:**
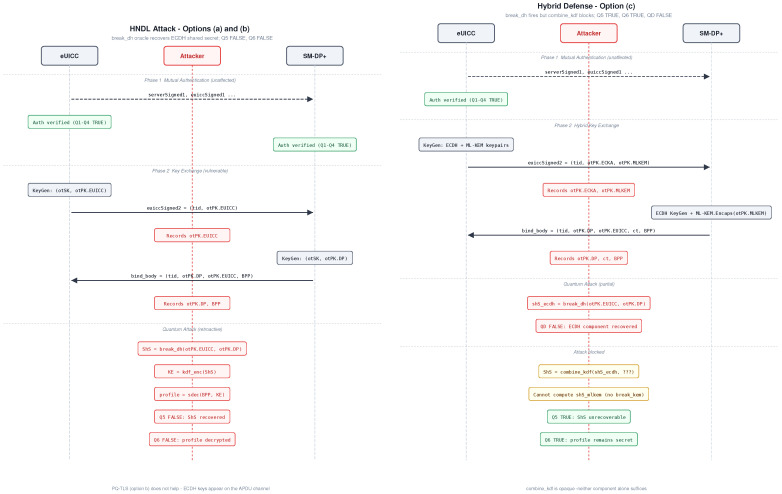
ProVerif traces under the break_dh oracle. **Left** (config. (a)/config. (b)): The attacker harvests otPK.DP.ECKA and otPK.EUICC.ECKA, fires break_dh, derives $\left(K_{E},K_{M}\right)$, and decrypts Profile. **Right** (config. (c)): break_dh fires (QD = FALSE), but the derivation halts at combine_kdf because ${\mathrm{shS}}_{2}$ is MLWE-protected under A2. Green nodes are public-channel observations; red nodes are attacker-derived steps.

**Configurations (a) and (b).** Q5 and Q6 return FALSE. The attacker observes both ephemeral ECDH public keys on the APDU channel (present in BF38 and BF21 respectively, regardless of configuration), applies break_dh to recover shS, and derives $\left(K_{E},K_{M}\right)$ via the standard KDF (Figure 3, left). Config. (b) is *not* HNDL-resistant despite PQ-TLS on ES9+, because PQ-TLS protects the HTTPS envelope but not the APDU keys already in PC/SC plaintext. This protocol-specific result shows that transport-layer PQC does not protect the ephemeral ECDH values carried on the local-APDU path.
**Configuration (c).** Q5 and Q6 return TRUE while QD returns FALSE: the oracle fires and recovers ${\mathrm{shS}}_{1}$, but combine_kdf also requires ${\mathrm{shS}}_{2}$ from ML-KEM-768 decapsulation, which is not recoverable from ct alone under A2 (Figure 3, right). This verdict transposes the standard-model combiner results of Bindel et al. [33] and Giacon, Heuer, and Poettering [40] to the BF38/BF21/BF36 wire format; we do not claim it as a new primitive-level result, since the combiner survival follows definitionally from A6. The protocol-specific, non-trivial finding is the configuration-(a)/(b) FAIL (PQ-TLS on ES9+ alone leaves the ephemeral ECDH share exposed on the PC/SC bus), together with a mechanical check, instrumented with an oracle-fire diagnostic, that the wire format does not leak combiner inputs outside the abstraction. A symbolic Shor-oracle TRUE verdict certifies the absence of structural protocol attacks, not concrete bit-security; the computational grounding is made explicit below.

In the symbolic model the result is robust to the combiner variant. A companion model identical to configuration (c), except that the two-argument combine_kdf is replaced by an X-Wing-style five-argument combiner (adapted to SGP.22’s two ephemeral ECDH public keys),

$${\mathrm{ShS}}_{\mathrm{xwing}}=H\left(\mathrm{label}\;∥\;{\mathrm{shS}}_{2}\;∥\;{\mathrm{shS}}_{1}\;∥\;ct\;∥\;{\mathrm{otPK}}_{\mathrm{DP}}\;∥\;{\mathrm{otPK}}_{\mathrm{EUICC}}\right),\;H=\text{SHA3-256},$$

declared as fun xwing_combine(bitstring, bitstring, kemct, dhpkey, dhpkey): bitstring, reproduces every verdict (Table 8), namely Q1–Q7 TRUE and QD FALSE under the break_dh oracle, because the three extra inputs (ct and both ECDH public keys) are public and do not narrow the attack set. This is a symbolic-equivalence robustness check on the A6 PRF abstraction, not a computational reduction: X-Wing’s separate advantage, KDF-layer transcript binding against cross-component substitution, is already covered in SGP.22 by the bind_body signature (which itself degrades under break_ecdsa, Section 6.6). More generally, any combiner modeled as an opaque PRF under A6 yields identical verdicts, so the configuration-(c) HNDL result is invariant to the combiner choice in the symbolic model.

**Computational-security grounding.** The symbolic verdict is the image of a standard-model result rather than a free-standing claim. combine_kdf is a dual-input KEM combiner whose session-key indistinguishability follows in the standard model from the combiner theorems of Bindel et al. [33] and Giacon, Heuer, and Poettering [40] under the Indistinguishability under Adaptive Chosen-Ciphertext Attack (IND-CCA2) security of ML-KEM-768 (FIPS 203) and the hardness of ECDH, and Assumption A6 is exactly the PRF premise of that reduction. Specifically, the reduction is a two-step game hop. First, replacing the combine_kdf output with a uniform key is detectable only with the PRF-distinguishing advantage of A6. The residual advantage against the session key is then bounded by the IND-CCA2 advantage against ML-KEM-768, because the ECDH P-256 branch ${\mathrm{shS}}_{1}$ is already revealed under break_dh, and *K* remains indistinguishable as long as ${\mathrm{shS}}_{2}$ is unrecoverable, which instantiates the either-input combiner theorem of Bindel et al. [33]. The ProVerif verdict, therefore, certifies that the BF38/BF21/BF36 wire format does not leak combiner inputs outside this abstraction. It is not itself a computational post-quantum proof. The CryptoVerif and ProVerif analysis of Signal PQXDH [15] is the methodological precedent for closing the remaining gap, and a machine-checked CryptoVerif reduction of combine_kdf under MLWE against a quantum-equipped adversary is the natural complementary next step (Section 7).
**Configuration (d).** All queries return TRUE, and QD is vacuously inapplicable because no ECDH operation exists. Q8 (classical post-compromise secrecy) holds trivially in all four configurations because the model does not retain ephemeral keys. This is the standard ephemeral Diffie–Hellman (DH) forward-secrecy result, and we list it for completeness rather than as an independent HNDL verification result. A computational-security reduction of combine_kdf under MLWE, outside ProVerif’s symbolic scope, is left for future work with CryptoVerif.

### 4.6. Symbolic Model Encoding

To make the encoding concrete beyond the per-configuration processes of Algorithms 1–4, the configuration-(c) equational theory declares Existential Unforgeability under Chosen-Message Attack (EUF-CMA) signatures, the ECDH key agreement, the unconditional break_dh oracle, the IND-CCA2 ML-KEM (no break_kem rule, per A2), the symmetric and KDF primitives, and the eUICC step-27b recovery combine_kdf $\left({\mathrm{shS}}_{1},{\mathrm{shS}}_{2}\right)$; the X-Wing companion changes only the final combiner to a five-input xwing_combine that additionally binds the ML-KEM-768 ciphertext and both ephemeral ECDH public keys. The same scheme instantiates the four configurations and their classical, forward-secrecy, and break_ecdsa variants by substituting the corresponding primitives. The complete ProVerif source for all four configurations and their variants, from which these declarations are taken verbatim, is publicly released for independent verification (Data Availability Statement).

## 5. Empirical Findings

The empirical evidence has three distinct scopes. Platform A directly measures provisioning behavior and advertised free volatile memory on one sysmocom C2T research eUICC (sysmocom GmbH, Berlin, Germany). Platform B directly measures PQC cycles and memory on an STM32 Nucleo-F446RE development board (STMicroelectronics, Geneva, Switzerland). Configuration-level RAM totals and SC300 implications combine these measurements with buffer accounting and instruction-set analysis; they are derived or projected quantities rather than direct on-card PQC measurements.

### 5.1. eUICC and Cortex-M4 Deployment Testbed

We use two platforms separated by an Instruction-Set Architecture (ISA) boundary. Table 9 summarizes the hardware, firmware, and instrumentation. **Platform A** is a sysmocom C2T eUICC in a PC/SC reader, used for session-level wall-time measurements and live APDU capture. **Platform B** is an STM32 Nucleo-F446RE with cycle-accurate timer capture, used for PQC primitive benchmarks, where direct measurement on SC300 silicon is not possible because pqm4 assembly requires the ARMv7E-M DSP extension.

Platform A characterizes one SC300-based research card, while Platform B supplies controlled Cortex-M4F measurements. Neither platform characterizes the commercial eUICC market. Portable-C measurements test compatibility with the ARMv7-M instruction subset used by the SC300 target, whereas the assembly measurements require DSP instructions that are absent from that target; neither result substitutes for chip-faithful PQC timing on a commercial card.

**PC/SC calibration.** The staircase model t=α·b+β·n (Table 9) converts bind-body payload size into APDU overhead, with $R^{2}\;=\;0.99999$ across the primary calibration range. Configuration (c) lies in the model-supported small-payload range. For the larger 16,468 B config. (d) BF38 certificate-chain payload, this paper uses the direct high-range replay median of 97,097 μs. The model’s 88,594 μs prediction is retained only as a diagnostic for the T = 0 staircase direction.
**The ISA gap.** pqm4 assembly optimizations use UMULL and UMAAL ISA instructions extensively in the NTT butterfly. These execute in a single cycle on Cortex-M4F but are absent from SC300. Consequently, the pqm4 assembly path cannot target the tested SC300 core or another core lacking the required ARMv7E-M DSP instructions. PQClean portable-C uses only the ARMv7-M common subset and compiles for both targets. Its ratios are ISA-faithful but not chip-faithful because the SC300 and F446RE differ in memory wait states and bus timing. We therefore use those ratios as target-relevant projections, not measurements of SC300 execution; direct on-card PQC timing is part of the validation required in Section 6.5.

### 5.2. Platform A Classical Session Baseline

Table 10 summarizes per-APDU timing across 200 classical sessions. BF36 is the aggregate of 19 LoadBoundProfilePackage segments.

Two reference figures anchor all subsequent analyses. The *AES-BPP floor* is 13.839 s (53% of session). This is the dominant cost and creates the absorption buffer for PQC transmission overhead. The *volatile-RAM ceiling* is 9539 B free volatile memory, reported by the C2T’s GetEUICCInfo2.extCardResource.freeVolatileMemory Type–Length–Value (TLV) across 10 reads, which is the constraint tested in Section 6. The TLV is a GlobalPlatform card-level snapshot of free RAM at query time rather than a guaranteed per-applet quota. It bounds the additional allocation that an SGP.22 stack can request without forcing an allocation failure, which is the operational meaning relevant to the deployment argument below.

### 5.3. Finding A: PQC Compute Is Not the Bottleneck

It is commonly assumed that PQC imposes a heavier computational load than classical ECC on constrained eUICC hardware and that this is the principal deployment barrier. The measurement challenges this assumption in the deployment-relevant DSP-less regime. The headline result of Finding A is the deployment-relevant, per-decapsulation cycle ratio on DSP-less SC300-class silicon: compared with a portable-C ECDH P-256 baseline (mbedTLS generic-C), ML-KEM-768 decapsulation is 46.3× cheaper (a 0.0216× cycle ratio, Table 11), while on DSP-equipped Cortex-M4F with the best-known hand-tuned assembly, the two primitives are at rough parity. The structural mechanism behind the SC300 advantage is an ISA-independent multiply-instruction-count gap, detailed with the 174-fold per-innermost-multiply audit in Section 5.3.2 (Table 12); this 174-fold figure is a per-innermost-multiply count, and the commensurable per-decapsulation cycle ratios carry the deployment claim.

At the optimized assembly tier on Platform B, the old micro-ECC denominator overstates the Cortex-M4F advantage because this baseline is substantially slower than the best-known P-256 assembly. Re-basing to Emil Lenngren’s constant-time P256-Cortex-M4 implementation [47], measured on the same board and Data Watchpoint and Trace (DWT) methodology, gives 903,783 cycles (37.7 ms at 24 MHz) for ECDH shared-secret derivation, versus 743,393 cycles (31.0 ms) for ML-KEM-768 decapsulation. The result is near parity: ML-KEM-768 is 0.82× the ECDH cycle count for the decapsulation-versus-agreement step, or 1.22× faster.

The target-relevant comparison is the strict ARMv7-M portable tier. A re-compile of both primitives with -mcpu=cortex-m3, -march=armv7-m, -mthumb, and -mfloat-abi=soft forces the toolchain to emit only ARMv7-M baseline instructions. The newlib multilib selected by the linker is thumb/v7-m/nofp, and a static disassembly audit confirms zero UMAAL, SMLAxy, SMUAdx, SMLAdx, UQADD16, VLDR, VPUSH, VADD, VMUL, or other ARMv7E-M DSP/SIMD/VFP instructions across both firmware ELFs (Executable and Linkable Formats), including libc paths. On this Platform B build, PQClean ML-KEM-768 Decaps measures 2,161,932 cycles (90.1 ms at 24 MHz), versus 100,018,410 cycles (4167 ms) for the mbedTLS 3.6 generic-C ECDH P-256 agreement, a 0.0216× ratio, equivalent to a 46.3× speedup in favor of ML-KEM-768. The ECDH P-256 denominator is a generic-C reference rather than the best-known portable implementation, so this is a portable-reference comparison rather than an eUICC performance ratio. On DSP-equipped Cortex-M4F with the best-known assembly, the two primitives are near parity. Direct SC300 execution remains unmeasured.

#### 5.3.1. Cortex-M33 Deployment Target

The realistic Phase 2 silicon is the ARMv8-M mainline with the optional DSP extension (Cortex-M33 class: ST33 series, Thales SX900 series, IDEMIA Mira series). A parallel portable-C re-compile with -mcpu=cortex-m33 -march=armv8-m.main+dsp reproduces the strict-ARMv7-M ratio within 0.6% because GNU Compiler Collection (GCC) 13.2.1 does not auto-vectorize the cryptographic kernels with +dsp enabled. Only hand-written assembly exercises the DSP path. These ratios compare primitives, not the full hybrid step: the measured integrated config. (c) step-27b routine still costs 1.69× the implemented classical ECDH P-256 path, but the C2T session-level result is governed by the AES-BPP floor rather than by this primitive timing.

The Cortex-M4F result is consistent with the direction established by prior head-to-head reports (Table 1 in Section 2.2), including Saarinen’s energy ratio of 0.18× [23], wolfSSL’s time ratio of 0.31× [24], and Kannwischer’s large pqm4 cycle advantage on Cortex-M4 [20]. Re-basing the denominator to the best-known P-256 assembly changes the paper’s interpretation. However, on DSP-rich M4F silicon, ML-KEM is not an order of magnitude faster; it is near parity for the agreement/decapsulation step and slightly behind for the full eUICC-side key-generation-plus-decapsulation total. The deployment-relevant advantage is the strict-ARMv7-M portable-C regime.

#### 5.3.2. Mechanism: DSP Instruction Audit

The advantage has a structural origin. P-256 field multiplication decomposes a 256-bit product into an 8×8 limb grid with diagonal carry propagation, costing 174 UMULL/UMAAL instructions per field multiply (micro-ECC ASM). ML-KEM-768 NTT arithmetic over $q=3329<2^{14}$ fits intermediate products in 14 bits with no carry chain, so the pqm4 NTT butterfly costs 1–2 UMULL per coefficient multiply (Table 12).

**Audit methodology.** Each row of Table 12 is produced under the same toolchain family as Table 11. The ELF is disassembled with arm-none-eabi-objdump -d; inner-kernel symbol boundaries are extracted from the matching .lst listing; and UMULL/UMAAL/SMULL mnemonics inside those boundaries are counted. The counting unit is instructions per innermost multiply (ecc_native_mod_mul for ECC, ntt_butterfly for ML-KEM-768). These units are structurally incommensurable (one P-256 field multiply yields 256 bits of intermediate state, while one NTT butterfly yields a 14-bit coefficient), so 174-fold is a per-innermost-multiply statement rather than per Decaps. Per-Decaps propagation is reflected in the cycle ratios above.

The 174-fold per-innermost-multiply reduction is an instruction-level mechanism observed in the strict ARMv7-M comparison. It is not an on-card measurement or a per-session speedup. The measured cycle ratios above apply to Platform B; transferring them to the C2T requires chip-faithful validation.

### 5.4. Finding B: Descriptive PQ-TLS Session Timing on the Tested C2T Setup

#### 5.4.1. Session Elapsed Time

The 18–35× increase in PQC key material can increase transport and APDU costs. The tested software-AES C2T provides a descriptive loopback case in which the observed session means are close, while the network-shaped measurements below show that transport cost reappears on bandwidth-limited links. The dataset contains 200 classical sessions collected on 15 April 2026 and 200 PQ-TLS sessions collected on 16 April 2026 as separate sequential runs. The arms were not randomized or interleaved. Welch’s two-sample calculation [49] gives t=0.252, p=0.8012, and Cohen’s d=−0.025, but the observed serial dependence and run-day confounding mean that the nominal independence assumption and confidence-interval coverage are not ensured. We therefore report the mean difference and the Two One-Sided Test (TOST) calculations as descriptive sensitivity analyses, not as confirmatory evidence of equivalence (Table 13).

**Figure 4:**
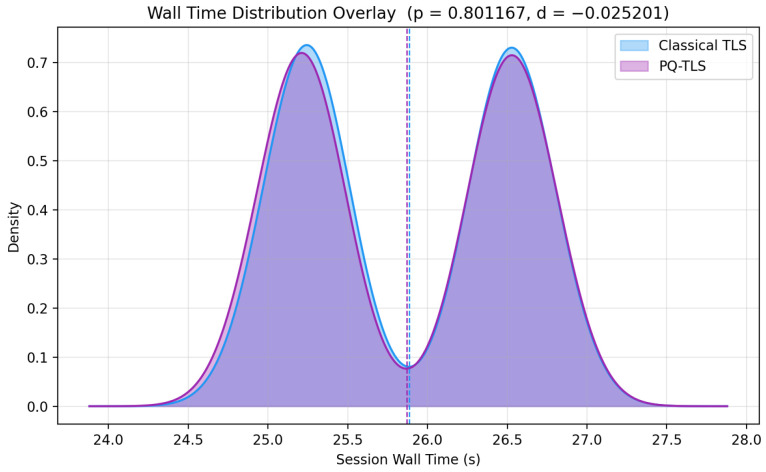
Descriptive loopback distributions for classical (config. (a), n=200) and PQ-TLS (config. (b), n=200) sessions on the tested C2T setup. The observed means differ by −0.017 s; Welch’s calculation gives t=0.252, p=0.8012, and Cohen’s d=−0.025. Exploratory TOST calculations pass at the tested ±0.5%, ±1%, and ±2% margins, but the arms were collected as separate sequential runs on different days and exhibit serial dependence. The figure therefore does not establish confirmatory equivalence. The network-shaped experiment is reported separately; hardware-AES cards also require new measurements.

**Exploratory margins.** Equivalence testing requires bounds tied to the smallest effect size of interest and preferably specified before outcome inspection [50]. We use ±1% of the classical session mean (here ±0.259 s) as an internal engineering margin derived from the measured session duration and the 13.839 s AES-BPP floor. It is not a preregistered or normative GSMA threshold. For transparency, we also calculate the results at ±0.5% and ±2%. The 90% Welch interval for the observed mean difference is [−0.128,+0.094] s and lies within all three tested margins. The confidence-bound geometry implies a crossover near ±0.4%, but this value is inferred rather than independently specified. These calculations show sensitivity to the chosen bound within the observed datasets; they do not remove the sequential-run confounding.
**Observed results and design limitations.** The classical mean is 25.886 s (SD 0.666 s), and the PQ-TLS mean is 25.869 s (SD 0.682 s), for an observed difference of −0.017 s. The released data show lag-1 correlations of −0.425 and −0.450 for the classical and PQ-TLS runs, respectively. Together with the separate collection days, this prevents a confirmatory causal or equivalence interpretation. Figure 4 therefore visualizes overlap in the two observed distributions, not interchangeability of TLS modes. A confirmatory follow-up should randomize or interleave the modes within the same acquisition window, prespecify the equivalence bound, and account for run order and host-bus state.
**Bimodal kernel-density structure.** Figure 4 depicts two modes in both arms. On the classical arm, a Hartigan dip test rejects unimodality (D=0.145, $p<10^{-4}$); the Sarle bimodality coefficient is 0.820, and a two-component Gaussian mixture is preferred over one (ΔBIC=322), with fitted components at 25.24 s and 26.54 s. A separate calibration that issues bare STORE DATA APDUs without SM-DP+, TLS, or PQC shows a similar structure, which is consistent with a host-transport contribution. It does not identify the latent state conclusively. Because the arms were collected sequentially, the bimodality and negative lag-1 correlation cannot be treated as harmless within-arm noise. The timing comparison therefore describes the combined eUICC, reader, host-bus, and collection-window behavior.

#### 5.4.2. Mechanism: The AES-BPP Decryption Floor

Within the tested C2T sessions, BF36 LoadBoundProfilePackage AES-128-CBC decryption consumes 13.839 s (53% of classical elapsed time). This large software-AES cost provides a plausible mechanism for the small observed difference between the sequential loopback runs. The PQ-TLS handshake is 7.85× larger than that of classical TLS (9270 vs. 1181 B, driven by the ML-DSA-44 CertificateVerify), while the separately measured handshake component differs by 0.50 ms. Because the full-session arms were collected on different days, the data do not causally isolate how much of the observed session difference is absorbed by the AES-BPP floor.

**Card-generation dependency.** The 13.839 s BF36 cost is a software-AES property of the C2T research card. A hardware-AES implementation may have a lower floor, making the projected configuration-(c) APDU overhead and the host-side PQ-TLS byte consume a larger fraction of session time. The magnitude cannot be inferred from this card. Both the session comparison and the engineering margin must therefore be re-estimated on each candidate card generation.

#### 5.4.3. APDU Payload Inflation

We use the PC/SC calibration model to convert bind-body growth into APDU overhead (Table 14).

Config. (c) adds 5 T = 0 blocks and 1269 B of payload for a total APDU overhead of 6.818 ms projected from the calibration model (0.0263% of session time). Config. (d) is dominated by the ML-DSA-44 certificate-chain payload and is directly measured at 97.1 ms for the BF38 transfer (0.3751% of session), and the calibration-model extrapolation of 88.6 ms is reported only as a diagnostic for the T = 0 staircase direction (see Table 14 caption, where * marks the directly measured value). A live APDU capture during a PQ-TLS session independently confirms that tag 5F49 (otPK.DP.ECKA, 65 B) appears in plaintext on the PC/SC bus, corroborating the formal result that config. (b) is not HNDL-resistant.

#### 5.4.4. Network-Shaped Sessions and Fleet Projection

The loopback equivalence result is not a general network result. The network-shaped session measurement re-introduces controlled round-trip time and bandwidth with tc netem and re-runs the real card-in-the-loop session harness, with n=30 classical and n=30 PQ-TLS sessions per condition. Table 15 reports the shaped-link result.

The shaped-link result refines Finding B rather than invalidating it. The shaped-link verdicts in Table 15 are *measured* (n=30 per arm), whereas the fleet-scale aggregates below are *projected* from the measured per-card APDU inflation. PQ-TLS adds bytes, not round trips: TLS 1.3 remains one round trip and ES9+ remains four. The additional ≈8 KB handshake remains TOST-equivalent at 10 Mbps/50 ms, becomes inconclusive at 10 Mbps/100 ms, and is statistically significant on the two 1 Mbps links. At the fleet scale, the ES10 APDU cost aggregates only on serial bulk or CI personalization lines: config. (c) adds +6.818 ms/card (+6.8 s per 1000 cards), and config. (d) adds +93.125 ms/card (+93 s per 1000 cards). For consumer over-the-air provisioning, the per-device network cost is local and parallel, while the shared SM-DP+ egress absorbs the additional PQ-TLS and full-PQ bytes.

### 5.5. Methodology Summary

The formal analysis uses ProVerif 2.05 [7,8] in the applied pi-calculus [9] under the unconditional break_dh equation, with a companion X-Wing variant as a symbolic check on the A6 PRF abstraction. Cycle benchmarks on Platform B use pqm4 [20], PQClean [21], micro-ECC [46], P256-Cortex-M4 [47], and mbedTLS 3.6 [48], reported as the median of 1000 trials per operation from the DWT counter. Session traces use lpac [41] over a localhost loopback at n=200 per arm with 10 warm-ups discarded; the PC/SC calibration fits 15 payload sizes at n=500 each, and the shaped-link runs use n=30 per arm. The two 200-session loopback arms were collected as separate sequential runs. Welch and TOST calculations are therefore reported descriptively, together with the collection-order and serial-dependence limitations. Table 16 maps each claim to its evidence class.

## 6. Discussion

### 6.1. Evidence Convergence: Selecting the Minimum HNDL-Safe Migration Step

The ProVerif model and the physical APDU capture are methodologically independent and converge on config. (c) as the minimum cryptographic-layer HNDL-resistant configuration within the four-configuration design space. The formal model proves Q5/Q6 = FAIL for config. (a) and config. (b), and the APDU capture confirms that both ephemeral ECDH public keys appear in PC/SC plaintext regardless of ES9+ transport. Table 16 consolidates the principal claims with their evidence classes.

### 6.2. Compute-and-Transmission Trade-Off

The compute comparison has two platform-bounded regimes. In the strict ARMv7-M portable-reference comparison on Platform B, ML-KEM-768 decapsulation is much cheaper than generic-C ECDH agreement; on the DSP-equipped Cortex-M4F with the best-known assembly, the two are near parity. The full hybrid step still runs ECDH and ML-KEM-768 sequentially. The C2T sessions also become larger in bytes: BF21 grows from 668 to 1937 B, and the ES9+ handshake grows from 1181 to 9270 B. The two sequential loopback runs have closely aligned observed means, but their collection design does not establish equivalence or show that the AES-BPP floor causally hides the added work. The independently shaped network experiment shows the byte cost reappearing on 1 Mbps links. The evidence therefore points to volatile RAM, payload size, and instruction-set support as deployment considerations, while session-time effects require a randomized same-window follow-up.

### 6.3. Volatile RAM Is the Binding Deployment Constraint

Within configuration-level resource accounting, volatile RAM is the binding feasibility constraint. Table 17 compares derived concurrent demand with the 9539 B C2T observation, separating fixed SGP.22 buffers from ML-KEM stack demand measured on Platform B.

Config. (c) therefore needs 12,451 B only for the optimized m4fspeed tier measured on Platform B, and the SC300-compatible PQClean portable-C tier raises the peak to 20,675 B. Both exceed the C2T’s 9539 B free-RAM ceiling, so no HNDL-resistant configuration fits on the tested card: the tiers are a requirement for higher-RAM silicon, not a property the C2T already satisfies. Config. (d) peaks at 29,712 B (3.11× the C2T ceiling), driven mainly by a 16,256-byte ML-DSA-44 certificate chain and an 8496-byte crypto working set, with the balance in stack and message buffers. The eUICC hardware requirements section of SGP.22 v3.1 [1] (§2.4 “eUICC and Profile Requirements” and the referenced SGP.21 Annex compliance items) constrains NVM, certificate-store, and profile-handling capabilities but does not specify a minimum free-volatile-RAM figure for the active provisioning session. As a result, a compliant SGP.22 eUICC may ship without sufficient RAM to execute the mainstream hybrid transitional migration (GSMA PQ.03 [35]). The specification currently provides no compliance mechanism to prevent this, and the present paper outlines a candidate mechanism in Section 6.4.

### 6.4. A Silicon-Tiered Phased Migration

Table 18 sketches a candidate three-phase migration path whose gating condition is available volatile RAM. The RAM figures are derived from the C2T baseline, STM32 implementation measurements, and protocol-buffer accounting. They are illustrative targets for validation, not values ready for adoption in SGP.22; the required cross-vendor evidence is set out in Section 7.

In this candidate path, Phase 1 uses config. (b) without closing HNDL because Q5 and Q6 fail under the oracle. Phase 2 would use config. (c) at the projected 16 KB optimized or 24 KB portable-C target, closing the HNDL window in the formal model with a projected 6.7 ms APDU overhead on the tested PC/SC calibration. Phase 3 would use config. (d) at the projected 32 KB target with streaming ML-DSA-44 certificate verification. These phases are design recommendations, not validated compliance tiers.

Operationally the tier is exposed as an eUICC capability rather than inferred by the LPA. During GetEUICCInfo2 (SGP.22 §5.7.8) the card advertises a PQC capability tuple (pqc-hybrid-16k-opt, pqc-hybrid-24k-portable, pqc-full-32k, with optional pqc-streaming-verify and pqc-hybrid-coproc). A [PRIVATE]-tagged extension inside the existing EUICCInfo2 Abstract Syntax Notation One (ASN.1) structure preserves backward compatibility. Legacy SM-DP+ implementations ignore the unknown tag under ASN.1 extensibility (the extension marker in the EUICCInfo2 module), with decoders skipping fields beyond the marker per the ASN.1 encoding rules, and fall back to config. (a) or config. (b). Binding the tuple under euiccSigned1 prevents an LPA-side downgrade because tuple substitution fails the outer signature check at the SM-DP+. Note that the binding requires an SM-DP+-side change to validate the tuple as authenticated input, not merely an LPA-side capability advertisement.

**Hardening scenario.** First-order masking has been demonstrated for ML-KEM on Cortex-M4 [51], but this study does not measure its cost on the C2T or STM32 configurations used here. A 30 to 50% increase is retained only as a literature-derived sizing scenario: applied to the unmasked peaks, it would yield 16.2 to 18.7 KB for the m4fspeed case and 26.9 to 31.0 KB for portable C. These values are projections and cannot establish a certified-card requirement.

### 6.5. Cross-Vendor Validation Required

The C2T measurements and STM32 benchmarks form a single-platform evidence chain, not a vendor-neutral compliance class. Public secure-element datasheets commonly report total or platform RAM rather than the applet-available volatile partition during provisioning, so the C2T observation cannot be placed reliably within the commercial distribution. The 16/24/32 KB entries are therefore validation targets, not numbers ratifiable in SGP.22. Before any target could be adopted, a cross-vendor study would need to measure peak RAM and AES-BPP cost for configurations (c) and (d) on multiple commercial eUICCs under their deployment toolchains and countermeasures, together with a representative profile-size survey. Hardware acceleration is another possible design path, but its cost and certification implications are not evaluated here. Vendor validation and certification would determine the schedule; this study does not estimate a deployment timeline.

### 6.6. The Authentication Horizon: From Hybrid Migration to Full PQC

When a live CRQC becomes available, ECDSA in config. (a)–config. (c) becomes forgeable, so the break_ecdsa analysis is the formal substrate of the Phase 3 (configuration (d)) recommendation, not an addendum. A scoped ProVerif re-run under a break_ecdsa(pk) → sk destructor returns Q1–Q4 and Q7 all FALSE for config. (a)–config. (c), with concrete forgery traces. The model is deliberately scoped (single non-replicated session, certificate hierarchy elided under the uncompromised-CI assumption (T1, Section 3.4), break_dh removed to isolate the oracle); eliding the hierarchy removes the four cert-reduction rules that otherwise blow up the Horn-clause resolver, and a false-preserving control (removing only the break_ecdsa equation returns Q1–Q4 and Q7 all TRUE) confirms that every FALSE verdict is attributable to the oracle alone. Under config. (d) the rule does not apply (ML-DSA-44 is MSIS-hard under quantum attack); Q1–Q4 and Q7 hold, and ML-DSA-44 verification is 0.226× ECDSA in cycles with 37.8× larger signatures, both absorbed by the AES-BPP floor. The combined break_dh + break_ecdsa verification on the full !-replicated process exhausted 14 GB without converging, so we handle the joint adversary by an explicit non-interference decomposition: the secrecy queries Q5/Q6 depend only on the KEM branch and combine_kdf and are invariant under break_ecdsa (which touches no rule on ${\mathrm{shS}}_{2}$ or the combiner), while the authentication queries Q1–Q4 and Q7 depend only on the signature layer and are invariant under break_dh. The joint verdict is thus the conjunction of the two separately machine-checked single-oracle verdicts, and the 14 GB non-convergence reflects a resolver blow-up rather than an open question, in the style of the component-wise PQXDH analysis [15]. The break_dh-only verdict remains meaningful because, under Mosca’s threshold, the key-exchange (HNDL) and signature-forgery horizons are temporally staged, configuration (c) closing the former and configuration (d) the latter. A unified single-model Tamarin re-encoding is a direction for future work.

#### CI Re-Personalization as the Operational Floor

The break_ecdsa result implies that config. (d) also requires a post-quantum trust-anchor path. One possible scenario is in-factory dual-rooted personalization with both ECDSA P-256 and ML-DSA-44 CI anchors; another is an authenticated field-update mechanism. The ML-DSA-44 verification key is 1312 B, compared with 64 B for ECDSA P-256, so either design adds non-volatile-memory and certificate-management costs. This study does not validate either operational path or estimate its rollout schedule. The discussion is a forward-looking consequence of the formal signature-forgery result, not deployment evidence.

### 6.7. Standards and Implementer Takeaways

For eSIM implementers, the formal result is that PQ-TLS on ES9+ alone does not close the HNDL window. Addressing the modeled exposure requires hybridizing step 27b (config. (c)) or replacing it (config. (d)). Resource accounting identifies volatile RAM as the binding constraint under the tested assumptions and motivates the illustrative 16/24/32 KB validation targets.

**Alternative encryption approaches.** Within the post-quantum design space, the algorithm slot admits substitutes that this study does not evaluate: the KpqC-selected SMAUG-T and NTRU+ KEMs, the code-based HQC, and a certificate-only hybrid pairing classical and post-quantum signatures. The integration pipeline is algorithm-agnostic and would re-run on any of these, and the X-Wing combiner is a natural alternative where KDF-internal transcript binding is required (Section 4.5). A fundamentally different paradigm, quantum key distribution (QKD), is not a good fit for SGP.22 consumer RSP: QKD secures a point-to-point link rather than the at-rest, store-and-forward Bound Profile Package that is the HNDL target; it requires dedicated optical or quantum channels and trusted-relay infrastructure that is absent from the LPA-to-eUICC PC/SC path and the constrained secure element, and it lies outside the SGP.22 and NIST/GSMA migration scope. A software-deployable PQC or hybrid migration, therefore, remains the only practical path for the over-the-air, constrained-device settings studied here.
A consolidated statement of the study’s limitations, and the future work they motivate, is given separately in Section 7.

## 7. Limitations and Future Work

The evidence in this study is deliberately scoped. We state its boundaries here, separately from the contributions, so that the results are not misread beyond what the data support and so that the caveats do not have to be repeated inline throughout the paper.

**Single-platform hardware scope.** The empirical evidence comes from one sysmocom C2T research eUICC and one STM32 board. C2T supplies the provisioning, AES-BPP, and advertised-RAM observations; STM32 supplies the PQC cycle and memory measurements. This is a platform-bounded existence study, not a vendor-neutral characterization of the eUICC market. The 9539 B observation and 13.839 s AES-BPP time are single-card values, while the 16/24/32 KB targets combine them with STM32 measurements and buffer accounting. Cross-vendor peak-RAM, AES-BPP, and direct on-card PQC measurements are required before any target can inform compliance.
**Session-comparison design and equivalence margins.** The 200 classical and 200 PQ-TLS sessions were collected as separate sequential runs on different days, not randomized or interleaved. Their negative lag-1 correlations and run-day confounding invalidate a confirmatory equivalence interpretation of the nominal Welch/TOST calculations. The ±1% margin is the internal engineering smallest effect size of interest derived from the C2T session duration and AES-BPP floor, not a preregistered or normative GSMA threshold. The ±0.5%, ±1%, and ±2% calculations are retained only as descriptive sensitivity analyses. A follow-up should randomize or interleave the arms within one acquisition window, prespecify the margin, and model the run order and host-bus state.
**Threat-model boundary and out-of-scope attacks.** The symbolic model covers protocol logic under a Dolev–Yao attacker extended with a quantum key-recovery oracle (break_dh) and, separately, a signature-forgery oracle (break_ecdsa). Physical-layer attacks, namely, side-channel analysis, fault injection, and physical key extraction, lie outside this model. We discuss them only to estimate their RAM and cycle costs for hardened implementations (Section 6.4); we make no claim of side-channel or fault resistance, and a leakage-model treatment of masked ML-KEM-768 and ML-DSA-44 under a formal side-channel adversary is left for future work. The long-term migration and Certificate Issuer re-personalization discussion (Section CI Re-Personalization as the Operational Floor) is likewise a forward-looking roadmap grounded in the break_ecdsa re-run, not an empirically validated deployment.
**Formal-model scope.** The ProVerif verdicts are symbolic: a TRUE verdict certifies the absence of structural protocol attacks under the stated assumptions A1–A6 (Table 4), not concrete bit-security. The configuration-(c) HNDL result follows from the pseudorandom-function abstraction A6 and transposes established combiner theorems; we give its computational grounding as a game-hop argument in Section 4.5, but a machine-checked CryptoVerif reduction of combine_kdf under MLWE against a quantum-equipped adversary remains future work. The combined break_dh + break_ecdsa verification on the fully replicated process exhausted 14 GB without converging, so we handle the joint adversary by explicit non-interference decomposition in Section 6.6, and a unified single-model Tamarin re-encoding is a direction for future work.
**Measurement and extrapolation scope.** The cycle counts in Table 11 are toolchain-strict re-compiles on the F446RE; they do not reproduce the memory hierarchy, security transitions, clocking, or firmware of a commercial eUICC. Direct on-card latency must be measured on each target. APDU payloads larger than 2636 B lie outside the primary PC/SC calibration, so configuration (d) uses a direct high-range replay. The loopback session comparison is descriptive for the tested software-AES setup and does not transfer across card generations.
**Future work.** Beyond the items above, the natural next steps are cross-vendor RAM, AES-BPP, and session measurements on commercial NXP, Infineon, and ST eUICCs; the CryptoVerif and Tamarin analyses noted above; a masked, side-channel-hardened evaluation of on-eUICC ML-KEM-768 and ML-DSA-44; and porting the construction to the GSMA SGP.32 (Internet of Things) and SGP.02 (machine-to-machine) profiles, whose 10-to-20-year device lifetimes widen the HNDL harvest window further.

## 8. Conclusions

Consumer eSIM provisioning will outlive the security of the classical cryptography that protects it today, so migrating SGP.22 to post-quantum cryptography is a matter of when, not whether. The paper’s main contribution is an end-to-end, resource-aware PQC migration framework for consumer RSP, not a new cryptographic primitive. It connects a protocol-specific HNDL analysis to a candidate configuration choice, its derived RAM requirements, and a safe mixed-fleet rollout mechanism.

The central practical message is that PQ-TLS alone does not close the HNDL window: the vulnerable key exchange remains exposed on the local-APDU link beneath the transport layer. Our formal analysis identifies hybrid configuration (c), classical ECDH P-256 combined with post-quantum ML-KEM-768, as the lightest option that resists HNDL, while the scoped signature-forgery analysis identifies fully post-quantum configuration (d) as the eventual authentication-safe end state.

Deployment evidence makes this framework actionable. In the tested STM32 analysis, post-quantum key exchange is not the binding compute cost; under the combined C2T, STM32, and buffer-accounting assumptions, volatile RAM is the deployment constraint. We quantify each configuration’s demand, translate it into candidate RAM tiers, and propose capability negotiation so that an operator can select a secure configuration for each authenticated, advertised card capability. Together, the formal verdicts, research-card provisioning observations, STM32 PQC measurements, RAM accounting, and negotiation design provide developers and implementers of standards with a concrete, evidence-based path from vulnerable SGP.22 provisioning to quantum-safe consumer RSP. Cross-vendor and direct on-card PQC measurements remain necessary before the candidate tiers can support commercial deployment or standardization.

## Figures and Tables

**Table 1:** Independent head-to-head studies of ML-KEM-768/Kyber against ECDH P-256 on the same hardware. Units vary by row (^a^ mJ, ^b^ ms, ^d^ kcycles). The PQ primitive is lighter in all four.

Source (Microcontroller Unit, MCU; Clock)	ML-KEM-768/Kyber	ECDH P-256	Ratio (PQ/EC)
Saarinen [23] (M4, 96 MHz)	2.57 ^a^	14.5 ^a^	0.18×
wolfSSL [24] (M4, 168 MHz)	5.64 ^b^	18.14 ^b^	0.31×
Kannwischer et al. [20] (M4)	n/a	n/a	large cycle advantage
**This work** (M4F, 24 MHz)	**743** ^d^	**904** ^d^	**0.82×**

Bold marks this study’s own measurement row.

**Table 2:** Scope of the closest prior work and the residual contribution of this study. The table distinguishes protocol scope and evidence type rather than ranking the studies.

Source	Primary Scope	Evidence Supplied	Outside That Source’s Scope
Ahmed et al. [16]	Consumer SGP.22 RSP	Applied pi-calculus model and ProVerif analysis under classical and partial-compromise scenarios	Quantum key recovery, PQC resource costs, and RAM-aware negotiation
Bettale et al. [19]	PQ secure channels for eSIM application management	ProVerif/Verifpal models and chip-based runtime and bandwidth measurements	SGP.22 profile-protection key agreement and local-APDU HNDL
Damir et al. [14]	Post-quantum 5G-AKA	ProVerif analysis and communication/computation costs	Consumer RSP, eUICC provisioning, and capability negotiation
Bindel et al.; Giacon et al. [33,40]	Generic hybrid-KEM construction	Computational definitions and combiner theorems	SGP.22 wire format and device feasibility
Embedded PQC libraries [20,21]	Embedded PQC implementations	Cortex-M benchmarking and portable-C implementations	RSP protocol analysis and eUICC provisioning behavior
GSMA PQ.03 [35]	Telecom PQC migration guidance	Use-case and migration recommendations	SGP.22-specific proof and device-grounded RAM analysis
**This work**	Consumer SGP.22 PQC migration	Quantum-oracle ProVerif model, C2T provisioning/RAM observations, STM32 PQC measurements, and resource projections	Commercial cross-vendor validation and direct on-card PQC timing

**Table 3:** Key and ciphertext sizes for elliptic-curve cryptography (ECC) against PQC. PQC values are from FIPS 203/204 [25,26]. In the SGP.22-role column, otPK.EUICC.MLKEM is the eUICC’s ML-KEM public (encapsulation) key, serverSigned1 is the SM-DP+-signed structure carrying the ML-KEM ciphertext, and serverSignature1 is the SM-DP+ authentication signature.

Material	Size (B)	vs. ECC	SGP.22 Role
ECDH P-256 public key	65	1.0×	BF38/BF21 key exchange
ML-KEM-768 encap. key	1184	18.2×	BF21 otPK.EUICC.MLKEM (config. (c), config. (d))
ML-KEM-768 ciphertext	1088	16.7×	BF38 serverSigned1 (config. (c), config. (d))
ECDSA P-256 signature	64	1.0×	BF38 authentication
ML-DSA-44 signature	2420	37.8×	BF38 serverSignature1 (config. (d))

**Table 4:** Computational assumptions and the part of the verdict each one gates.

ID	Assumption	Role in the Verdict
A1	ECDH P-256 is classically hard, inverted only by the break_dh oracle (Equation (1))	Defines the harvest oracle and the config. (a)/config. (b) FAIL
A2	MLWE is hard classically and quantumly (no break_kem oracle)	Gates config. (c) Q5/Q6 survival and the QD = FALSE reading
A3	MSIS is hard classically and quantumly (no break_sign oracle)	Gates config. (d) authentication (Q1–Q4, Q7) under ML-DSA-44
A4	SHA-256 and SHA3-256 behave as random oracles	Idealizes the hashing and key-derivation primitives
A5	AES-128 is semantically secure	Frames the inherited AES-128 BPP Grover floor
A6	KDF and combine_kdf are pseudorandom functions (PRFs)	Gates config. (c) combiner survival and the combiner-agnostic Q5/Q6 result

**Table 5:** Four SGP.22 post-quantum migration configurations. HNDL status: Section 4; RAM: Section 6.

Config.	APDU Key Exchange	ES9+	HNDL	Peak RAM
(a)	ECDH P-256	TLS 1.3	**Vuln.**	2643 B
(b)	ECDH P-256	PQ-TLS (ML-KEM-768)	**Vuln.**	2643 B
(c)	ECDH P-256 ‖ ML-KEM-768	PQ-TLS (ML-KEM-768)	**Resistant**	12,451 B opt., 20,675 B portable-C
(d)	ML-KEM-768 (+ML-DSA-44 auth)	PQ-TLS (ML-KEM-768)	**Resistant**	29,712 B

**Table 6:** Security queries verified by ProVerif (Q1–Q8) plus the oracle diagnostic (QD), under the break_dh-extended Dolev–Yao attacker.

ID	Property	Informal Statement
Q1	eUICC auth.	SM-DP+ verifies eUICC identity
Q2	Server auth.	eUICC verifies SM-DP+ identity
Q3	Mutual auth.	Q1 and Q2 hold simultaneously
Q4	Session integ.	Session params. not modified in transit
Q5	Key secrecy	$\left(K_{E},K_{M}\right)$ indistinguishable from uniform
Q6	Profile confid.	BPP ciphertext not invertible
Q7	Bind integrity	Profile bound to correct eUICC Identifier (EID)
Q8	Classical post-compromise secrecy (PCS)	Past keys safe after long-term signing-key exposure
QD	Oracle diag.	attacker(shS_1): is the ECDH-branch secret recoverable?

**Table 7:** ProVerif results under the break_dh oracle. **Fail** = property violated. QD = FALSE means the oracle fired and recovered ${\mathrm{shS}}_{1}$.

Property	(a)	(b)	(c)	(d)
Q1–Q4 (mutual auth.)	Pass	Pass	Pass	Pass
Q5 (session-key secrecy)	**Fail**	**Fail**	**Pass**	Pass
Q6 (BPP confidentiality)	**Fail**	**Fail**	**Pass**	Pass
Q7 (binding integrity)	Pass	Pass	Pass	Pass
Q8 (classical PCS)	Pass	Pass	Pass	Pass
QD (oracle diagnostic)	N/A	N/A	**Fires (blocked)**	N/A

**Table 8:** Verdict comparison of the primary combine_kdf model and the X-Wing companion model under the break_dh oracle. The verdicts agree on every property, so HNDL resistance of configuration (c) is robust to the combiner choice in the symbolic model.

Property	combine_kdf	xwing_combine
Q1–Q4 (mutual auth.)	Pass	Pass
Q5 (session-key secrecy)	Pass	Pass
Q6 (BPP confidentiality)	Pass	Pass
Q7 (binding integrity)	Pass	Pass
QD (oracle diagnostic)	Fires (blocked)	Fires (blocked)

**Table 9:** Measurement environment. The 9539 B figure is GetEUICCInfo2.extCardResource.freeVolatileMemory on the C2T (10/10 reads), a single-card ceiling.

Parameter	Value
*Platform A (eUICC session measurement)*	
eUICC	sysmocom C2T research eUICC; ARM SC300 (Arm Ltd., Cambridge, UK; ARMv7-M Thumb-2, *no* DSP); free volatile RAM 9539 B
Host/reader/SM-DP+	x86-64 Linux + LPA client; HID Omnikey 3x21 Universal Serial Bus (USB) reader (HID Global, Austin, TX, USA); osmo-smdpp localhost (wolfSSL 5.7)
APDU timing	per-tag ns-resolution process timestamps
ES9+ transport	TLS 1.3 (config. (a)) or PQ-TLS/ML-KEM-768 (config. (b)); n=200 per config. (10 warm-ups discarded)
*Platform B (Cortex-M4F cycle benchmarks)*	
Board/core/clock	STM32 Nucleo-F446RE, ARM Cortex-M4F, 24 MHz, zero flash wait states (Data Watchpoint and Trace, DWT, cycle counter; SysTick-verified)
ISA	ARMv7E-M (Thumb-2 + DSP: UMULL, UMAAL, SMULL, SMLAL); bare metal, interrupts disabled
PQC/ECC	pqm4 m4fspeed (assembly, ASM) [20], PQClean portable-C [21], micro-ECC ASM [46], P256-Cortex-M4 [47], mbedTLS 3.6 generic-C [48]
Trials	1000 per operation; median reported (coefficient of variation, CV, annotated for ML-DSA-44 sign)
*PC/SC calibration & tooling*	
Calibration	15 sizes 16–2636 B, n=500 each; model t=αb+βn, b=⌈n/255⌉; α=1040.4μs/block, β=1.27μs/byte, $R^{2}=0.99999$
Statistics	Welch two-sample *t*-test [49] + Cohen’s *d*; CLOCK_MONOTONIC (host)/DWT (STM32)
Formal	ProVerif 2.05 [7,8] applied pi-calculus, break_dh destructor

**Table 10:** Platform A session timing summary (n=200, config. (a)). BF36 aggregates 19 LoadBoundProfilePackage segments.

Operation (Tag)	Median (µs)	Std (µs)	CV	% Session
BF38 AuthenticateServer	3,112,246	102,969	0.033	12.0
BF21 PrepareDownload	3,749,426	459,396	0.118	14.5
BF36 LoadBPP (aggregate)	13,839,000	N/A	N/A	53.5
ES9+ initiateAuth.	24,304	5151	0.208	0.09
ES9+ authenticateClient	33,982	5840	0.169	0.13
ES9+ getBoundProfilePkg	27,554	4729	0.174	0.11
**Total elapsed time**	25,886,000	666,000	0.026	100

**Table 11:** Platform B (Cortex-M4F) cycle counts and latencies at the 24 MHz zero-wait-state measurement clock. The Cortex-M4F assembly rows use the best-known P-256 assembly for the ECDH denominator and pqm4 m4fspeed for ML-KEM. The strict-ARMv7-M and ARMv8-M-DSP rows report SC300-faithful and Cortex-M33-target portable-C re-compiles, whose cryptographic kernels emit zero DSP/SIMD/VFP instructions in a static disassembly audit. The hybrid row reports the measured integrated config. (c) step-27b routine.

Op.	Tier/Target	Impl.	Cycles ($\times 10^{3}$)	Time (ms)	Ratio/Baseline
*ARMv7E-M assembly tier (Cortex-M4F + DSP)*					
ECDH P-256 agree	M4F asm	P256-Cortex-M4 [47]	904	37.7	1.00×
ML-KEM-768 KeyGen	M4F asm	pqm4 m4fspeed	664	27.7	0.73×
ML-KEM-768 Encaps	M4F asm	pqm4 m4fspeed	694	28.9	0.77×
ML-KEM-768 Decaps	M4F asm	pqm4 m4fspeed	743	31.0	**0.82×**
ML-DSA-44 KeyGen	M4F asm	pqm4 m4fspeed	1360	56.7	n/a
ML-DSA-44 Sign	M4F asm	pqm4 m4fspeed (CV 0.73)	2910	121.2	n/a
ML-DSA-44 Verify	M4F asm	pqm4 m4fspeed	1440	60.0	n/a
*Strict ARMv7-M portable-C tier (SC300-faithful, zero DSP audited)*					
ECDH P-256 agree	v7-M no-DSP	mbedTLS 3.6 generic-C	100,018	4167	1.00×
ML-KEM-768 Decaps	v7-M no-DSP	PQClean portable-C	2162	90.1	**0.0216×**
*ARMv8-M-DSP portable-C tier (Cortex-M33 target)*					
ECDH P-256 agree	v8-M+dsp	mbedTLS 3.6 generic-C	99,780	4157	1.00×
ML-KEM-768 Decaps	v8-M+dsp	PQClean portable-C	2145	89.4	**0.0215×**
Hybrid step-27b	M4F asm	integrated asm routine	11,539	480.8	1.69× impl. path

The Strict-ARMv7-M build uses -mcpu=cortex-m3, -march=armv7-m, -mthumb, and -mfloat-abi=soft. The ARMv8-M-DSP build uses -mcpu=cortex-m33, -march=armv8-m.main+dsp, -mthumb, and -mfloat-abi=soft. Both were audited with arm-none-eabi-objdump -d to emit zero UMAAL/SMLAxy/SMUAdx/SMLAdx/UQADD16/VFP/SIMD instructions in the cryptographic kernels. Bold marks the headline ML-KEM-versus-ECDH ratios.

**Table 12:** Per-innermost-multiply audit: UMULL/UMAAL counts within one field multiply (ECC) or one NTT butterfly (ML-KEM, ML-DSA). Units are structurally incommensurable per row (256-bit vs. 14-bit intermediate), so the 174-fold ratio is a per-innermost statement holding on any ARMv7-M including SC300.

Algorithm	Implementation	UMULL/UMAAL per Innermost Multiply
ECDH P-256	mbedTLS (generic C)	11
ECDH P-256	micro-ECC (M4 ASM) [46]	174
ML-KEM-768	PQClean (portable C) [21]	1
ML-KEM-768	pqm4 m4fspeed (ASM) [20]	2
ML-DSA-44	PQClean (portable C) [21]	2
ML-DSA-44	pqm4 m4fspeed (ASM) [20]	93

**Table 13:** Descriptive session wall-time comparison on Platform A: config. (a) vs. config. (b), n=200 sequentially collected sessions per arm.

Metric	Classical	PQ-TLS
Mean elapsed time (s)	25.886	25.869
Standard deviation (s)	0.666	0.682
TLS handshake bytes	1181	9270
TLS handshake latency (ms)	8.5	9.0
Welch *t*-statistic	0.252	
*p*-Value	0.8012	
Cohen’s *d*	−0.025	
90% confidence interval for mean difference	[−0.128,+0.094] s	
Exploratory TOST margin	±1% session time (±0.259 s), bound passed	
Handshake as % of session	<0.05%	

**Table 14:** APDU bind-body sizes and T = 0 overhead per configuration. Configs. (a)/(c) are PC/SC model projections within the calibrated range. * Config. (d) is the direct high-range BF38 replay (97,097 μs), and the 88,594 μs model extrapolation is retained only as a diagnostic.

Cfg.	APDU	Payload (B)	T = 0 blks	Overhead (µs)	% Session
(a)	BF21	668	3	3972	0.015
(c)	BF21	1937	8	10,790	0.0417
(d)	BF38	16,468	65	97,097 *	0.375
config. (c) additional vs. config. (a):			+5 blks	+6818 μs	+0.0263
config. (d) additional vs. config. (a):			+62 blks	+93,125 μs	+0.3598

**Table 15:** PQ-TLS session wall-time delta under tc netem loopback shaping, n=30 per arm per condition. Verdict pairs Welch’s two-sided test at α=0.05 with the TOST outcome at the ±0.5 s margin.

Condition	Classical (s)	PQ-TLS (s)	Δ (s)	Welch *p*	Verdict
10 Mbps/50 ms (LTE)	26.976	27.144	+0.168	0.357	equivalent
10 Mbps/100 ms (HSPA+)	27.727	28.050	+0.323	0.083	inconclusive
1 Mbps/50 ms (DSL)	27.348	27.717	+0.369	0.043	significant
1 Mbps/100 ms (UMTS)	28.124	28.642	+0.518	0.006	significant

**Table 16:** Consolidated evidence for principal claims. Proven denotes ProVerif under stated assumptions; Measured denotes a direct platform observation; Derived denotes arithmetic composition of measured components; Projected denotes a model-based extrapolation; Recommended denotes a design or standards proposal.

Claim	Class	Basis
config. (a)/config. (b) fail HNDL secrecy under break_dh	Proven	ProVerif + APDU trace
config. (c) preserves secrecy after ECDH compromise	Proven	ProVerif + X-Wing variant
Observed loopback mean difference, PQ-TLS minus classical, is −0.017 s	Measured	sequential C2T runs; descriptive only
PQ-TLS shaped-link: equivalent at LTE, significant at ≤1 Mbps	Measured	netem, n=30/arm, Welch + TOST
config. (c) APDU inflation 6.818 ms	Projected	PC/SC calibration $R^{2}=0.99999$
Fleet-scale ES10 APDU cost (+6.8/+93 s per 1000 cards)	Projected	composition of measured APDU inflation
ML-KEM-768.Decaps 46.3× faster in the strict ARMv7-M portable-reference comparison; near parity with best-known M4F assembly	Measured	Platform B + static audit
RAM totals 12,451/20,675/29,712 B against the 9539 B C2T observation	Derived	STM32 stack + protocol-buffer accounting
Candidate 16/24/32 KB RAM tiers (single-card)	Recommended	C2T baseline, pending Section 6.5

**Table 17:** Config. (c) hybrid volatile-RAM sensitivity. Non-stack terms total 6195 B (3648 B key material + 32 B long-term ECDSA + 2515 B four-cert ECDSA chain) before ML-KEM stack.

ML-KEM Tier	SC300 Compat.	Stack	Peak	Minimum Tier
pqm4 m4fstack memory-opt.	No	2844 B	9039 B	current/16 KB
pqm4 m4fspeed local	No	6256 B	12,451 B	16 KB
pqm4 m4fspeed upstream	No	6452 B	12,647 B	16 KB
PQClean portable-C	Yes	14,480 B	20,675 B	24 KB

**Table 18:** Candidate three-phase SGP.22 PQ migration. The RAM entries are illustrative single-platform targets derived from C2T and STM32 evidence and remain pending cross-vendor validation (Section 6.5). Hardening overhead is not included in the table.

Phase	Config.	Illustrative RAM Target	HNDL-Safe
1 (baseline)	(b) PQ-TLS only	measured C2T: <16 KB	No
2a (hybrid-opt.)	(c) ECDH P-256 + ML-KEM-768	projected 16 KB	**Yes**
2b (hybrid-port.)	(c) ECDH P-256 + ML-KEM-768	projected 24 KB	**Yes**
3 (full PQC)	(d) ML-KEM-768 + ML-DSA-44	projected 32 KB	**Yes**

## Data Availability

The complete ProVerif source models for the four migration configurations (a)–(d), including the classical baselines, the break_dh quantum-oracle scenarios, the X-Wing companion model, the forward-secrecy variants, and the scoped break_ecdsa signature-forgery re-run, together with the raw ProVerif output logs and a script that reproduces every reported formal-model verdict, are publicly available at https://github.com/Lavelliane/pq-rsp/releases/tag/v1.0 (accessed on 20 July 2026; commit 281abef92a242dddd693f10eb40243ec52281c81).

## Abbreviations

The following abbreviations are used in this manuscript:

3GPP	3rd Generation Partnership Project
AES	Advanced Encryption Standard
AKA	Authentication and Key Agreement
APDU	Application Protocol Data Unit
ASN.1	Abstract Syntax Notation One
AVX2	Advanced Vector Extensions 2
BAN	Burrows–Abadi–Needham
BIC	Bayesian Information Criterion
BPP	Bound Profile Package
CBC	Cipher Block Chaining
CCID	Chip Card Interface Device
CCRA	Common Criteria Recognition Arrangement
CI	Certificate Issuer
CRQC	Cryptographically Relevant Quantum Computer
CSPRNG	Cryptographically Secure Pseudorandom Number Generator
CV	Coefficient of Variation
DSP	Digital Signal Processing
DWT	Data Watchpoint and Trace
EAL	Evaluation Assurance Level
ECC	Elliptic-Curve Cryptography
ECDH	Elliptic-Curve Diffie–Hellman
ECDSA	Elliptic Curve Digital Signature Algorithm
EID	eUICC Identifier
ELF	Executable and Linkable Format
eSIM	Embedded SIM
EUF-CMA	Existential Unforgeability under Chosen-Message Attack
eUICC	Embedded Universal Integrated Circuit Card
EUM	eUICC Manufacturer
FIPS	Federal Information Processing Standards
GCC	GNU Compiler Collection
GSMA	GSM Association
HKDF	HMAC-based Key-Derivation Function
HNDL	Harvest-Now–Decrypt-Later
HTTPS	Hypertext Transfer Protocol Secure
IETF	Internet Engineering Task Force
IND-CCA2	Indistinguishability under Adaptive Chosen-Ciphertext Attack
IoT	Internet of Things
ISA	Instruction-Set Architecture
KDF	Key-Derivation Function
KEM	Key Encapsulation Mechanism
LPA	Local Profile Assistant
M2M	Machine-to-Machine
MCU	Microcontroller Unit
ML-DSA	Module-Lattice-Based Digital Signature Algorithm
ML-KEM	Module-Lattice-Based Key Encapsulation Mechanism
MLWE	Module Learning With Errors
MSIS	Module Short Integer Solution
NIST	National Institute of Standards and Technology
NTT	Number Theoretic Transform
NVM	Non-Volatile Memory
PC/SC	Personal Computer/Smart Card
PCS	Post-Compromise Secrecy
PKI	Public-Key Infrastructure
PQC	Post-Quantum Cryptography
PQ-TLS	Post-Quantum Transport Layer Security
PRF	Pseudorandom Function
QKD	Quantum Key Distribution
RAM	Random Access Memory
RSP	Remote SIM Provisioning
SAS	Security Accreditation Scheme
SM-DP+	Subscription Manager Data Preparation
TLS	Transport Layer Security
TLV	Type–Length–Value
TOSTs	Two One-Sided Tests
UICC	Universal Integrated Circuit Card
USB	Universal Serial Bus

## References

[1] GSMA (2023). SGP.22: RSP Technical Specification, Version 3.1.

[2] Shor P.W. (1999). Polynomial-Time Algorithms for Prime Factorization and Discrete Logarithms on a Quantum Computer. SIAM Rev..

[3] CISA, NSA, NIST Quantum-Readiness: Migration to Post-Quantum Cryptography. Joint Cybersecurity Information Sheet, CISA/NSA/NIST, 2023. https://www.cisa.gov/resources-tools/resources/quantum-readiness-migration-post-quantum-cryptography.

[4] Chen L., Jordan S., Liu Y.K., Moody D., Peralta R., Perlner R., Smith-Tone D. (2016). NISTIR 8105: Report on Post-Quantum Cryptography.

[5] Mosca M. (2018). Cybersecurity in an Era with Quantum Computers: Will We Be Ready?. IEEE Secur. Priv..

[6] Dolev D., Yao A. (1983). On the Security of Public Key Protocols. IEEE Trans. Inf. Theory.

[7] Blanchet B. An Efficient Cryptographic Protocol Verifier Based on Prolog Rules. Proceedings of the 14th IEEE Computer Security Foundations Workshop.

[8] Blanchet B. (2016). Modeling and Verifying Security Protocols with the Applied Pi Calculus and ProVerif. Found. Trends Priv. Secur..

[9] Abadi M., Fournet C. (2001). Mobile Values, New Names, and Secure Communication. Proceedings of the 28th ACM SIGPLAN-SIGACT Symposium on Principles of Programming Languages.

[10] Cremers C., Horvat M., Hoyland J., Scott S., van der Merwe T. (2017). A Comprehensive Symbolic Analysis of TLS 1.3. Proceedings of the 2017 ACM SIGSAC Conference on Computer and Communications Security.

[11] Rescorla E. (2018). The Transport Layer Security (TLS) Protocol Version 1.3.

[12] Basin D., Dreier J., Hirschi L., Radomirović S., Sasse R., Stettler V. (2018). A Formal Analysis of 5G Authentication. Proceedings of the 2018 ACM SIGSAC Conference on Computer and Communications Security.

[13] Hussain S.R., Echeverria M., Karim I., Chowdhury O., Bertino E. (2019). 5GReasoner: A Property-Directed Security and Privacy Analysis Framework for 5G Cellular Network Protocol. Proceedings of the 2019 ACM SIGSAC Conference on Computer and Communications Security.

[14] Damir M.T., Meskanen T., Ramezanian S., Niemi V. (2022). A Beyond-5G Authentication and Key Agreement Protocol. Proceedings of the 16th International Conference, NSS 2022.

[15] Bhargavan K., Jacomme C., Kiefer F., Schmidt R. (2024). Formal Verification of the PQXDH Post-Quantum Key Agreement Protocol for End-to-End Secure Messaging. Proceedings of the 33rd USENIX Conference on Security Symposium.

[16] Ahmed A.S., Peltonen A., Sethi M., Aura T. (2024). Security Analysis of the Consumer Remote SIM Provisioning Protocol. ACM Trans. Priv. Secur..

[17] Ko Y., Lastre J.K., Kwon H., You I. (2026). Revisiting the M2M Remote SIM Provisioning Protocol: A Comprehensive Security and Performance Analysis. Alex. Eng. J..

[18] Lastre J.K., Ko Y., Kwon H., Kim B., You I. (2025). Formal Verification of Consumer Remote SIM Provisioning Common Mutual Authentication using BAN Logic. Proceedings of the 1st International Conference on Consumer Technology (ICCT-Pacific).

[19] Bettale L., Dottax E., Grémy L. Post-Quantum Secure Channel Protocols for eSIMs. Proceedings of the 22nd International Conference on Security and Cryptography.

[20] Kannwischer M.J., Rijneveld J., Schwabe P., Stoffelen K. pqm4: Testing and Benchmarking NIST PQC on ARM Cortex-M4. https://eprint.iacr.org/2019/844.

[21] PQClean Contributors (2024). PQClean: Clean, Portable, Tested Implementations of Post-Quantum Cryptography. https://github.com/PQClean/PQClean.

[22] Greconici D.O.C., Kannwischer M.J., Sprenkels D. (2021). Compact Dilithium Implementations on Cortex-M3 and Cortex-M4. IACR Trans. Cryptogr. Hardw. Embed. Syst..

[23] Saarinen M.J.O. (2020). Mobile Energy Requirements of the Upcoming NIST Post-Quantum Cryptography Standards. Proceedings of the IEEE International Conference on Mobile Cloud Computing, Services, and Engineering (MobileCloud).

[24] wolfSSL Benchmarks for Kyber Level 1 PQM4 Integration on STM32 ARM Cortex-M4. wolfSSL Benchmark Report. 7 December 2022 (Updated 2024). https://www.wolfssl.com/benchmarks-kyber-level-1-pqm4-integration-stm32-arm-cortex-m4/.

[25] NIST (2024). FIPS 203: ML-KEM Standard.

[26] NIST (2024). FIPS 204: ML-DSA Standard.

[27] NIST (2024). Transitioning the Use of Cryptographic Algorithms and Key Lengths.

[28] NIST (2024). Transition to Post-Quantum Cryptography Standards.

[29] NIST (2025). Recommendations for Key-Encapsulation Mechanisms.

[30] Barker E., Chen L., Davis R. (2020). Recommendation for Key-Derivation Methods in Key-Establishment Schemes.

[31] GPP (2024). Study on the Support of 256-bit Algorithms for 5G.

[32] Bos J.W., Costello C., Naehrig M., Stebila D. (2015). Post-Quantum Key Exchange for the TLS Protocol from the Ring Learning with Errors Problem. Proceedings of the IEEE Symposium on Security and Privacy.

[33] Bindel N., Brendel J., Fischlin M., Goncalves B., Stebila D. (2019). Hybrid Key Encapsulation Mechanisms and Authenticated Key Exchange. Proceedings of the 10th International Conference, PQCrypto 2019.

[34] Westerbaan B., Stebila D. (2023). X25519Kyber768Draft00 Hybrid Post-Quantum Key Agreement. IETF Internet Draft. https://www.douglas.stebila.ca/research/papers/draft-tls-westerbaan-xyber768d00/.

[35] GSMA (2024). PQ.03: Post-Quantum Cryptography Guidelines for Telecom Use Cases.

[36] GSMA (2025). PQ.04: Post Quantum Cryptography in IoT Ecosystem.

[37] Ko Y., Pawana I.W.A.J., You I. (2025). 5G-AKA-HPQC: Hybrid Post-Quantum Cryptography Protocol for Quantum-Resilient 5G Primary Authentication with Forward Secrecy. arXiv.

[38] Lastre J.K., Ko Y., Kwon H., You I. (2025). Evaluating Transport Layer Security 1.3 Optimization Strategies for 5G Cross-Border Roaming: A Comprehensive Security and Performance Analysis. Sensors.

[39] Kumar S., Al-Muhammed M.J., Vimal S., Jararweh Y. (2024). A Review of Lightweight Security and Privacy for Resource-Constrained IoT Devices. Comput. Mater. Contin..

[40] Giacon F., Heuer F., Poettering B. (2018). KEM Combiners. Proceedings of the 21st IACR International Conference on Practice and Theory of Public-Key Cryptography (PKC 2018).

[41] lpac Contributors (2024). lpac: C-Based eUICC LPA. https://github.com/estkme-group/lpac.

[42] PC/SC Workgroup (2023). Interoperability Specification for ICCs and Personal Computer Systems. https://pcscworkgroup.com/.

[43] Security Explorations (2025). Security Weaknesses of eSIM/eUICC Technology. https://security-explorations.com/esim-security.html.

[44] Barbosa M., Connolly D., Duarte J.D., Kaiser A., Schwabe P., Varner K., Westerbaan B. (2024). X-Wing: The Hybrid KEM You’ve Been Looking For. https://eprint.iacr.org/2024/039.

[45] Cremers C., Dax A., Medinger N. (2024). Keeping Up with the KEMs: Stronger Security Notions for KEMs and Automated Analysis of KEM-based Protocols. Proceedings of the ACM SIGSAC Conference on Computer and Communications Security (CCS).

[46] MacKay K. micro-ECC: A Small and Fast ECDH and ECDSA Implementation for 8-bit, 32-bit, and 64-bit Processors. 2014–2024. https://github.com/kmackay/micro-ecc.

[47] Lenngren E. (2021). P256-Cortex-M4: Constant-Time P-256 ECDSA/ECDH for ARM Cortex-M4 and Cortex-M33. https://github.com/Emill/P256-Cortex-M4.

[48] Arm Limited (2024). Mbed TLS: An Open Source, Portable, Easy to Use, Readable and Flexible TLS Library. https://github.com/Mbed-TLS/mbedtls.

[49] Welch B.L. (1947). The Generalization of ‘Student’s’ Problem When Several Different Population Variances are Involved. Biometrika.

[50] Lakens D. (2017). Equivalence Tests: A Practical Primer for t Tests, Correlations, and Meta-Analyses. Soc. Psychol. Personal. Sci..

[51] Heinz D., Kannwischer M.J., Land G., Pöppelmann T., Schwabe P., Sprenkels D. (2022). First-Order Masked Kyber on ARM Cortex-M4. https://eprint.iacr.org/2022/058.pdf.

